# Photodynamic therapy for prostate cancer: Recent advances, challenges and opportunities

**DOI:** 10.3389/fonc.2022.980239

**Published:** 2022-09-23

**Authors:** Qin Xue, Jingliang Zhang, Jianhua Jiao, Weijun Qin, Xiaojian Yang

**Affiliations:** Department of Urology, Xijing Hospital, Fourth Military Medical University, Xi’an, China

**Keywords:** photodynamic therapy, prostate cancer, photosensitizer, nanoparticle photosensitizers, clinical studies

## Abstract

Over the past two decades, there has been a tendency toward early diagnosis of prostate cancer due to raised awareness among the general public and professionals, as well as the promotion of prostate-specific antigen (PSA) screening. As a result, patients with prostate cancer are detected at an earlier stage. Due to the risks of urine incontinence, erectile dysfunction, etc., surgery is not advised because the tumor is so small at this early stage. Doctors typically only advise active surveillance. However, it will bring negative psychological effects on patients, such as anxiety. And there is a higher chance of cancer progression. Focal therapy has received increasing attention as an alternative option between active monitoring and radical therapy. Due to its minimally invasive, oncological safety, low toxicity, minimal effects on functional outcomes and support by level 1 evidence from the only RCT within the focal therapy literature, photodynamic treatment (PDT) holds significant promise as the focal therapy of choice over other modalities for men with localized prostate cancer. However, there are still numerous obstacles that prevent further advancement. The review that follows provides an overview of the preclinical and clinical published research on PDT for prostate cancer from 1999 to the present. It focuses on clinical applications of PDT and innovative techniques and technologies that address current problems, especially the use of nanoparticle photosensitizers in PDT of prostate cancer.

## Introduction

Prostate cancer is the second most common malignancy in men. In 2020, there will be 1.41 million new cases of prostate cancer worldwide and roughly 380 thousand fatalities, predicts the International Agency for Research on Cancer (IARC) of the World Health Organization (WHO) (1). Additionally, while there are fewer diagnosed cases of prostate cancer in Asia than in Europe or the United States, there is still a constant increase in the incidence of the disease (2, 3). Its biological parameters and prognosis differ substantially between individuals as a result of its significant variability. The stages of the disease determine treatment recommendations. Radical prostatectomy (RP), external beam radiation therapy (EBRT), together with androgen deprivation therapy (ADT) are curative alternatives for patients with locally advanced prostate cancer. However, adverse effects including erectile dysfunction and persistent incontinence have a significant impact on patients’ quality of survival. Such treatments are obviously inappropriate, especially for low-risk prostate cancer with smaller tumors that only take up 5-10% of the prostate volume and a higher propensity for unifocal or unilateral illness (4). Since prostate-specific antigen (PSA) screening became widely used during the past 20 years, the number of patients with low-risk prostate cancer has substantially increased. Patients in this group face a dilemma in that they can either select radical prostate therapy, which has a risk of decreased quality of life, or defer treatment (active surveillance/watchful waiting), which carries a higher chance of cancer progression (5). Other techniques, like cryotherapy, high-intensity focused ultrasound (HIFU), photodynamic therapy (PDT), and electroporation, have come into focus as potential therapeutic alternatives. These focal therapies have been developed as minimally invasive procedures. By sparing the neurovascular bundles, sphincter, and urethra, the goal is to selectively ablate tumors while minimizing damage (6, 7). PDT is palliative, repeatable, and low toxicity in comparison to other focal therapies. Moreover, the equipment is less expensive and takes up less room. Furthermore, PDT has access to sufficient information to support some initial conclusions. PDT has been used to treat cancers in patients for more than 40 years. It has demonstrated good safety and efficacy in treating a variety of malignancies, including skin, head, and neck cancers, etc (8). However, efficiency of PDT is constrained by the physiological features of prostate cancer. The potential of PDT for prostate cancer has once more been demonstrated with the introduction of vascular-targeted Tookad^®^. In the latest EAU guidelines 2022, the strength rating for focal treatment in a clinical trial setting for low- and intermediate-risk prostate cancer is strong (9). PDT will continue to be rigorously improved to fulfill its full promise as a prostate cancer treatment, despite its current shortcomings and uncertainty.

## Photodynamic therapy

### The mechanisms of PDT

**Figure 1:**
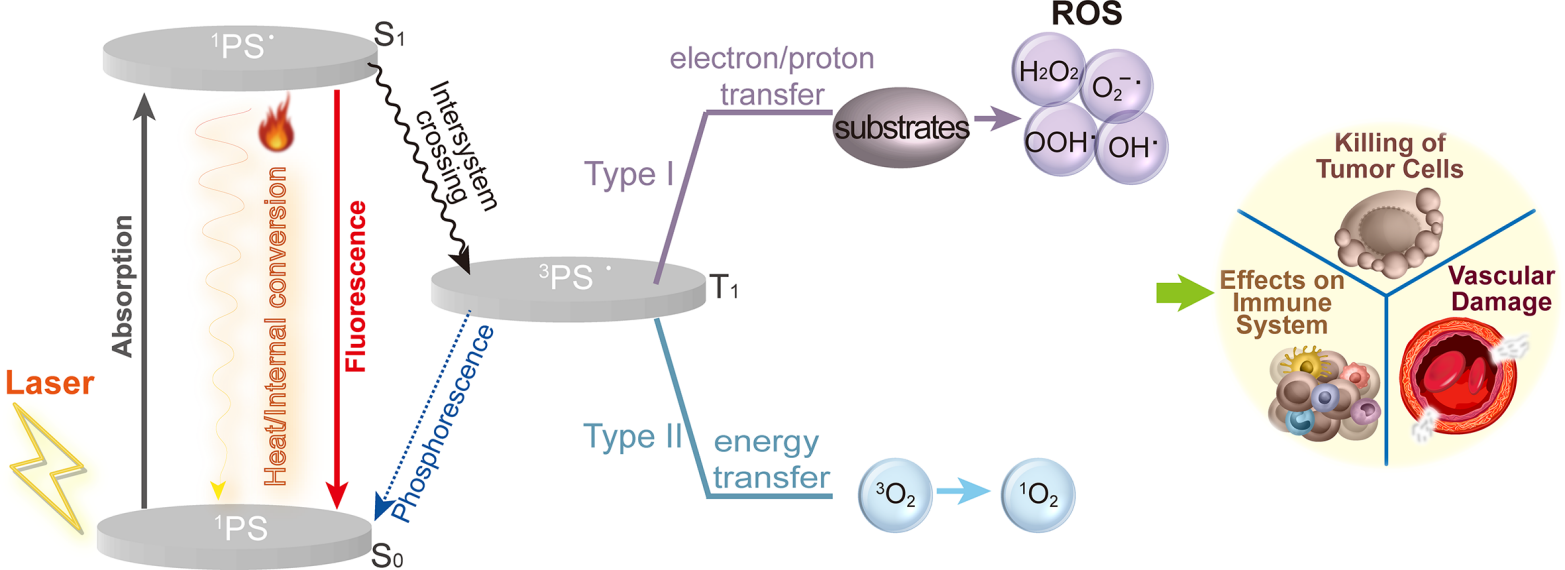
A generalized diagram depicting PDT for prostate cancer. PS was administered intravenously to patients at various times before LED excitation (depending on the type and dose of PS). The optical fibers are placed within plastic catheter needles that are positioned and guided by transrectal ultrasound and a brachytherapy-type template to the prostate gland *via* the perineum.

Since Schell extracted hematoporphyrin from dried blood in 1841 and Lipson and Schwartz used a hematoporphyrin derivative for fluorescence for tumor localization diagnosis in 1960 (10), PDT has rapidly advanced as a disease treatment modality. A photosensitizer (PS), light and reactive oxygen species (ROS) are the three primary components required by PDT to eliminate tumors (11). As shown in **Figure 1**, the photodynamic response involves both photophysical and photochemical reactions. When exposed to an absorption spectrum at a particular wavelength, PS absorbs photons and converted from the ground state (S_0_) to the excited singlet state (S_1_) as ^1^PS^•^, It subsequently releases its energy through illumination or internal conversion to heat. A photophysical energy emission, such as heat and fluorescence, may also accompany the excitation of ^1^PS^•^ to the triplet state (T_1_) as ^3^PS^•^. Through two different mechanisms, ^3^PS^•^ instantly produces ROS. According to the type I mechanism, PS directly interacts with substrates (such as the polyunsaturated fatty acids in the lipids of cell membrane) and produces organic radicals by transferring electrons or protons. Further reactions between the free radicals and cellular oxygen result in ROS like peroxides (H_2_O_2_, ROOH), superoxide anion (O_2_-^•^), hydroxyl radical (HO^•^) and hydroxyl radicals (HOO^•^). Type II involves the direct transfer of energy from the ^3^PS^•^ to triplet state oxygen (_3_O^2^) directly, followed by the formation of singlet oxygen (_1_O^2^) (12). When _1_O^2^ interacts with biomolecules such proteins, lipids, and nucleic acids, its primary products are hydroperoxides and endocyclic peroxides, which lead to a series of free radical peroxidation chain reactions (13). _1_O^2^ is considered to be the most significant ROS in PDT cytotoxicity. Both mechanisms can occur simultaneously. The PS type and concentrations as well as the availability of oxygen are just a few of the variables that may have an effect on the ratios between them. The activation of a series of molecular processes by photodynamic response ultimately leads in various forms of cell death, immune system impacts, and vascular damage (14, 15).

### Photosensitizers

As the most important part of PDT, numerous types of PSs have been developed. However, as indicated in **Table 1**, only a small number of them have received clinical therapeutic approval. The following qualities should be included in an ideal PS (1): strong photosensitization ability, i.e., high photochemical quantum yield; (2) high tumor targeting; (3) minimum dark toxicity and side effects; (4) biological stability and purity; (5) fast metabolism in normal tissues; (6) ideal hydrophilic properties. PSs can be categorized into first-generation and second-generation PSs based on when they were created. Generally, first-generation PSs refer to Porfimer sodium (Photofrin^®^) and Haematoporphyrin derivative (HpD, HiPorfin^®^), which were developed in the early 1980s and were the first to be approved for PDT of non-cutaneous solid tumors (16). In 1993, Photofrin^®^ was approved for the treatment of bladder cancer in Canada (17). Additional health authority authorization to treat various tumors came after this. The Food and Drug Administration (FDA) approved Photofrin^®^ for the treatment of esophageal cancer in 1995 and early non-small cell lung cancer in 1998, respectively. In 2001, HiPorfin^®^ was approved by National Medical Products Administration (NMDA, former China Food and Drug Administration) of China for bladder cancer, oesophageal cancer and lung cancer (18). Photofrin^®^ and HiPorfin^®^ have been used for many years by medical institutions around the world to treat tumors, and their efficacy and safety have been extensively acknowledged. However, it also has several significant drawbacks, including dark cytotoxicity, skin phototoxicity, complicated oligomeric composition, and limited absorption bands at red wavelengths. However, since the excitation light wavelength of the drug is 630 nm, a compromise that neither falls within the PS’s optimal absorption spectrum range nor within the tissue’s optimal light transmission wavelength range, the depth of the light penetration is constrained. Additionally, it stays in the skin for up to a few weeks, which might easily result in skin photosensitivity side effects. Consequently, since the late 1980s, second-generation PSs have been developed. Second-generation PSs are photosensitive compound monomers with high red-spectrum absorption, which reduces dark phototoxicity and improved penetration depth. Additionally, second-generation PSs are more quickly eliminated from healthy tissues than porphyrins, reducing skin phototoxicity. The majority of second-generation PSs, such as protoporphyrin IX (PpIX) precursors (e.g. ALA, Levulan^®^), chlorin (e.g. temoporfin, Foscan^®^) (19), bacteriochlorins (e.g. WST11, TOOKAD^®^), dye (e.g. phtalocyanine), etc., are derived from tetrapyrroles (20). **Table 1** lists the second-generation PSs that are currently approved: Levulan^®^ and its derivatives, methyl-ALA (Metvix^®^) and Hexaminolevulinate (Hexvix^®^), have received FDA, European Medicines Agency (EMA), NMDA, Sweden and Australia approval for the treatment of actinic keratoses, basal cell carcinoma, and bladder cancer detection. In Japan, Talaporfin (Laserphyrin^®^) was approved for the treatment of esophagus, lung, and brain tumors. Belarus has approved the use of Chlorin e6-PVP (Photolon^®^) for the diagnosis and treatment of skin and mucosal tumors. The EU and Mexico recently approved the use of TOOKAD^®^, a vascular-targeted padeliporfin, to treat prostate cancer. As shown in **Table 2**, a large number of PSs are undergoing clinical trials, and the commercialization and application prospects are quite promising. Curcumin, the hydrophobic polyphenol found in the rhizome of turmeric and Toluidine blue ortho have also been considered as potential PSs (21, 22). By adhering or introducing certain chemical compounds with biological properties to the structure of the second-generation PSs at the end of the 20th century, the third-generation of PSs were developed. Additionally, different drug delivery systems for PSs, such as micelles, liposomes, and hydrophilic polymers were developed. Almost all were still in the stage of animal trails.

**Table 1:** Examples of clinically approved PSs.

PSs	Chemical group	Approved year	Chemical structure		λ_max_	Approved applications	Approved countries
Porfimer sodium (Photofrin^®^)	Porphyrin	1993-1998	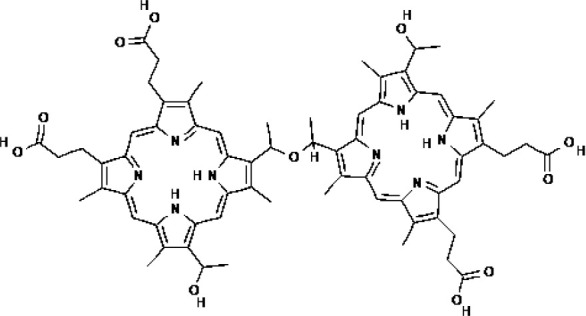		630 nm	Bladder cancer, Endobronchial cancer, Esophageal cancer, Cervical cancer, Lung cancer	USA, Canada, Japan, France, Netherlands, German, UK
Haematoporphyrin derivative (HpD, HiPorfin^®^)	Porphyrin	2001	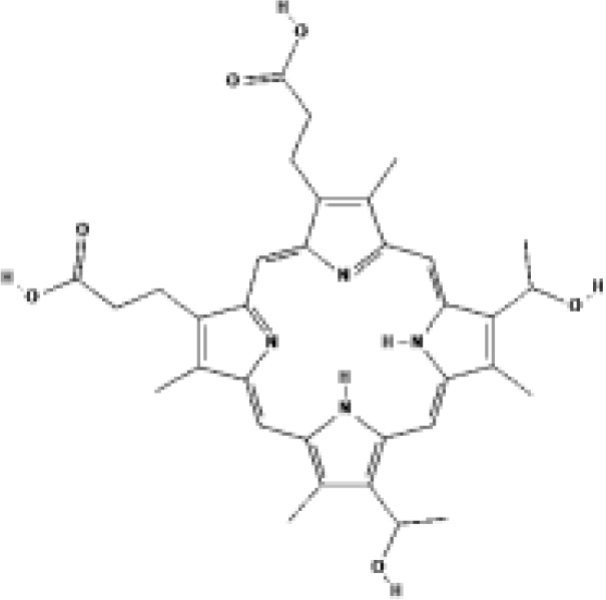		635 nm	Bladder cancer, Oesophageal cancer, Lung cancer	China
5-Aminolevulinic acid (5-ALA, Levulan^®^)	PpIX precursor	2017	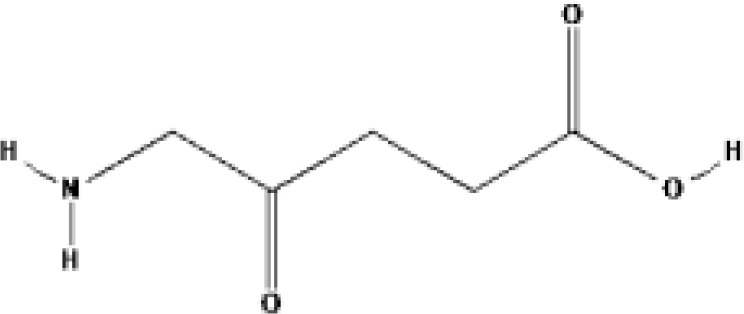		635 nm	Actinic keratoses, Basal cell carcinoma, Non-melanoma skin cancers, Squamous cell carcinoma	USA, UK
Methyl aminolevulinic acid (MAL, Metvix^®^)	PpIX precursor	2017	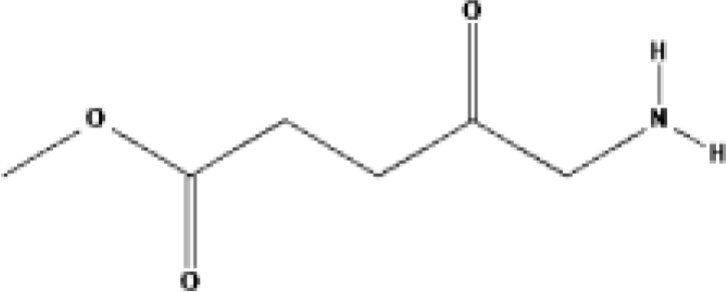		635 nm	Actinic keratoses, Basal cell carcinoma, Brain tumors diagnosis and guided resection	USA, UK, Australia
Hexaminolevulinate (HLA, Hexvix^®^)	PpIX precursor	2006	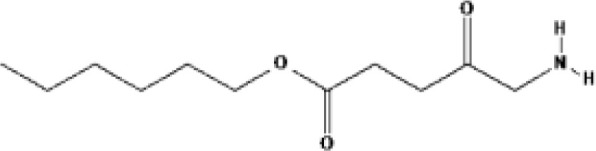		635 nm	Bladder cancer diagnosis	USA, EU, Sweden
	Protoporphyrin IX (PpIX)		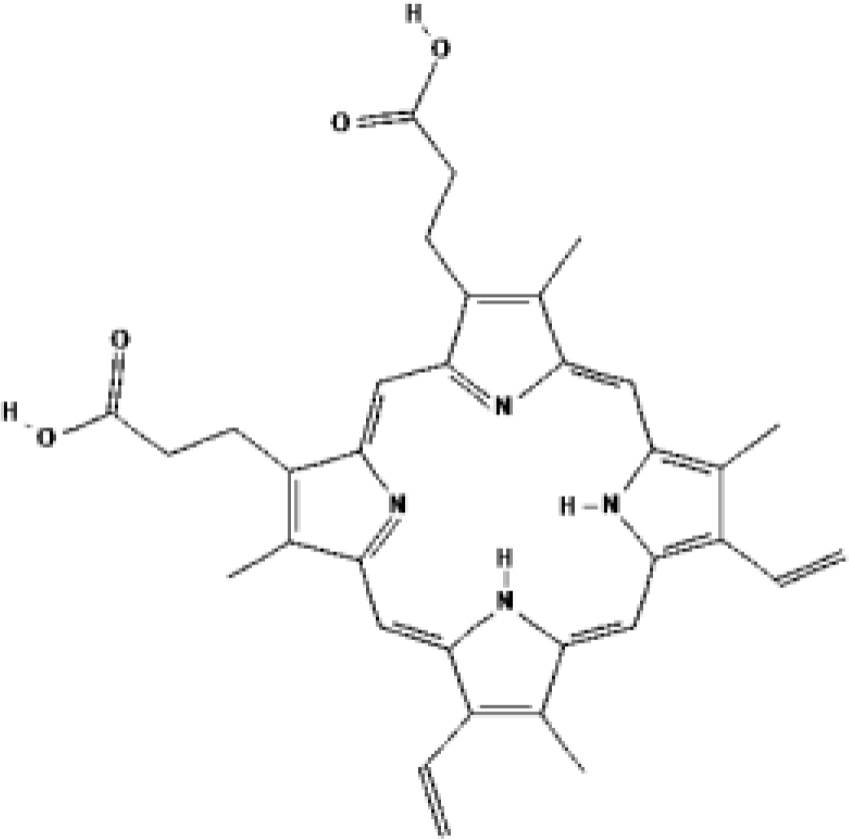				
Temoprfin (mTHPC, Foscan^®^)	Chlorin	2001	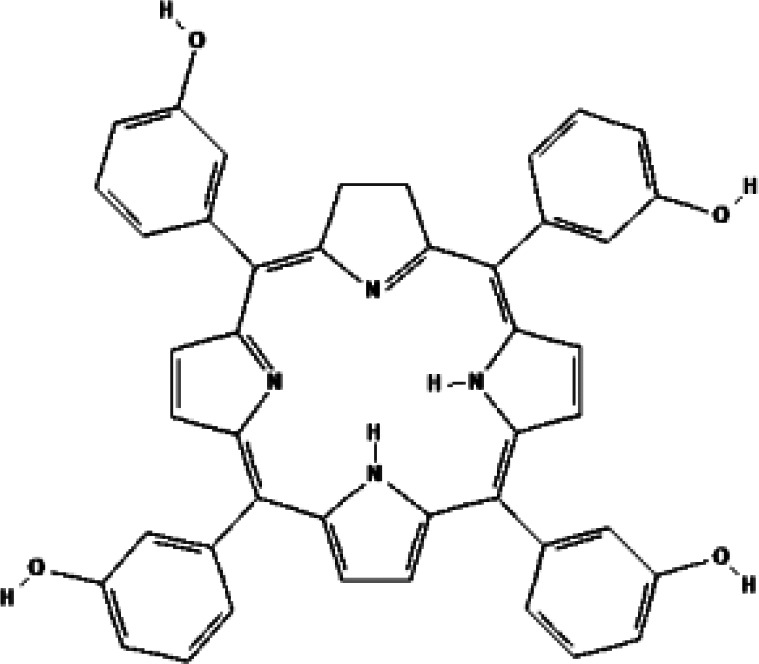		652 nm	Oesophageal cancer, Non-small cell lung cancer, Head and neck squamous cell carcinoma	EU, Norway, Iceland
Verteporfin (BPD-MA, Visudyne^®^)	Chlorin	2000	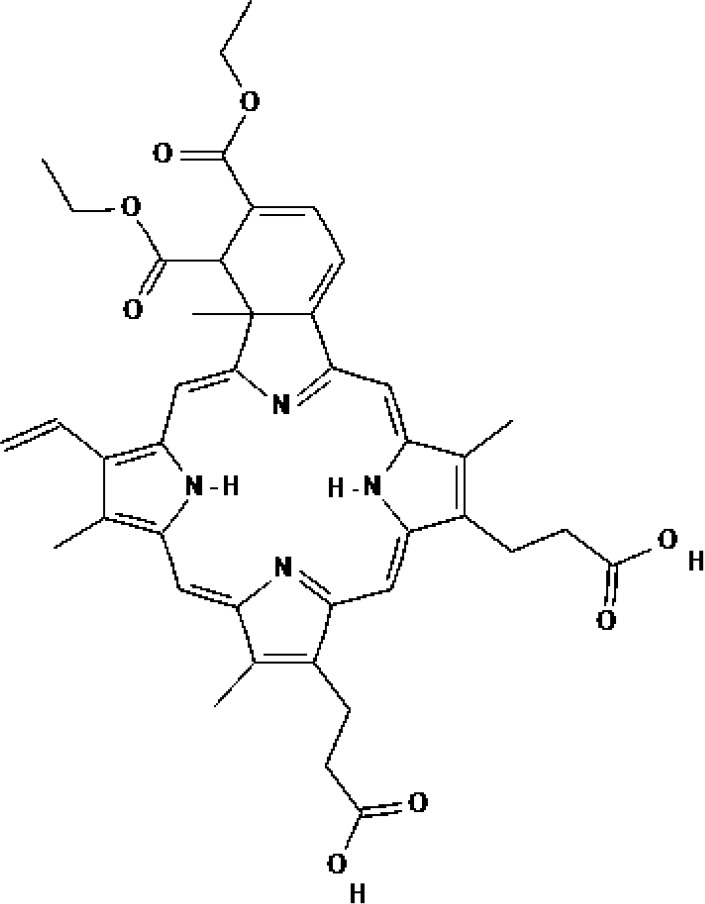		690 nm	Actinic keratoses	USA
Talaporfin (Npe6, Laserphyrin^®^)	Chlorin	2003	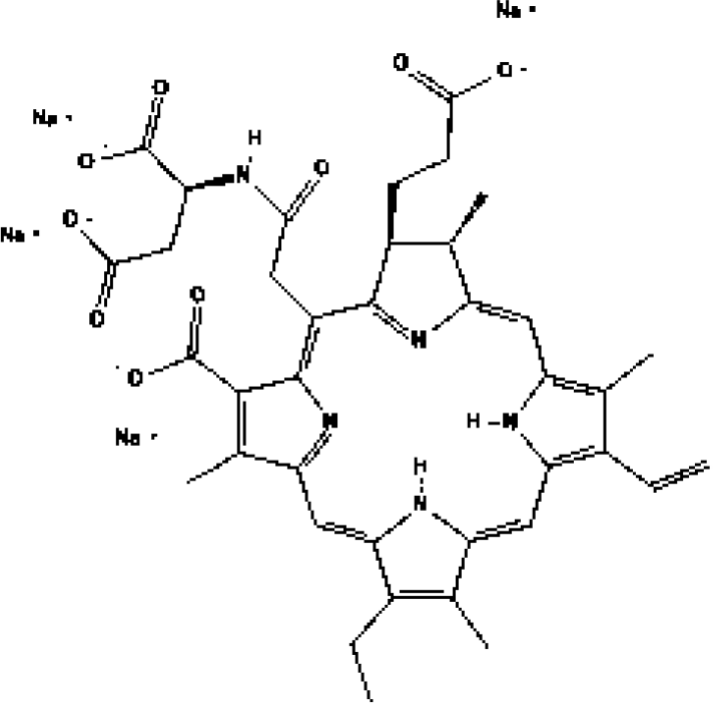		660 nm	Lung cancer, Brain tumor, Esophageal cancer	Japan
Padeliporfin (WST11, TOOKAD^®^)	Bacteriochlorin	2018	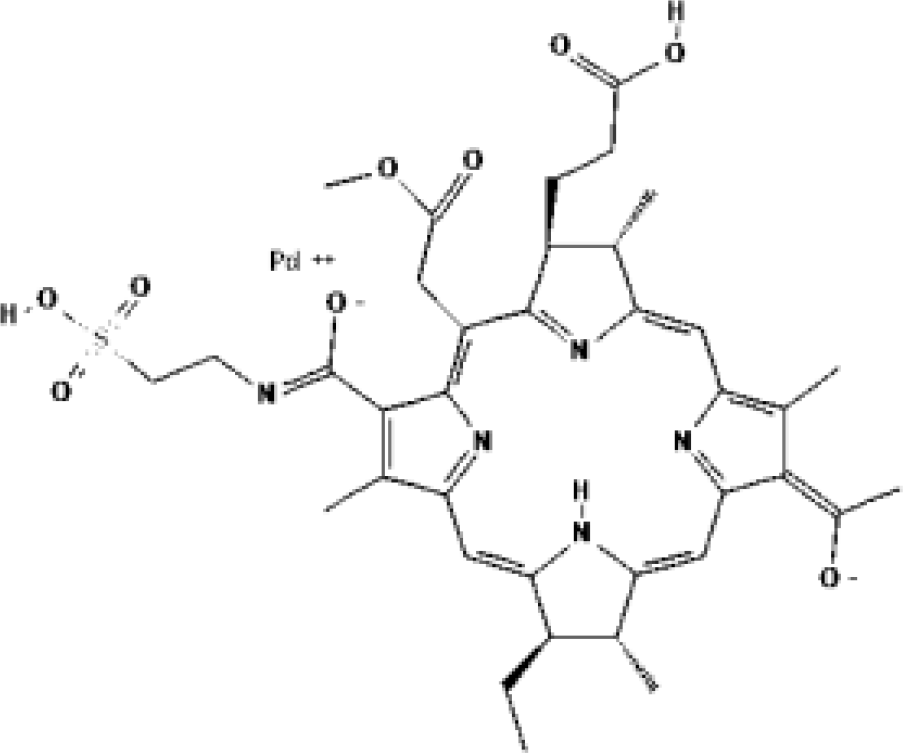		753 nm	Prostate cancer	Mexico, EU

**Table 2:** Examples of PSs in cancer clinical trials.

PSs	Chemical group	Chemical structure		λ_max_	Ongoing cinical trials
Porfimer sodium (Photofrin^®^)	Porphyrin	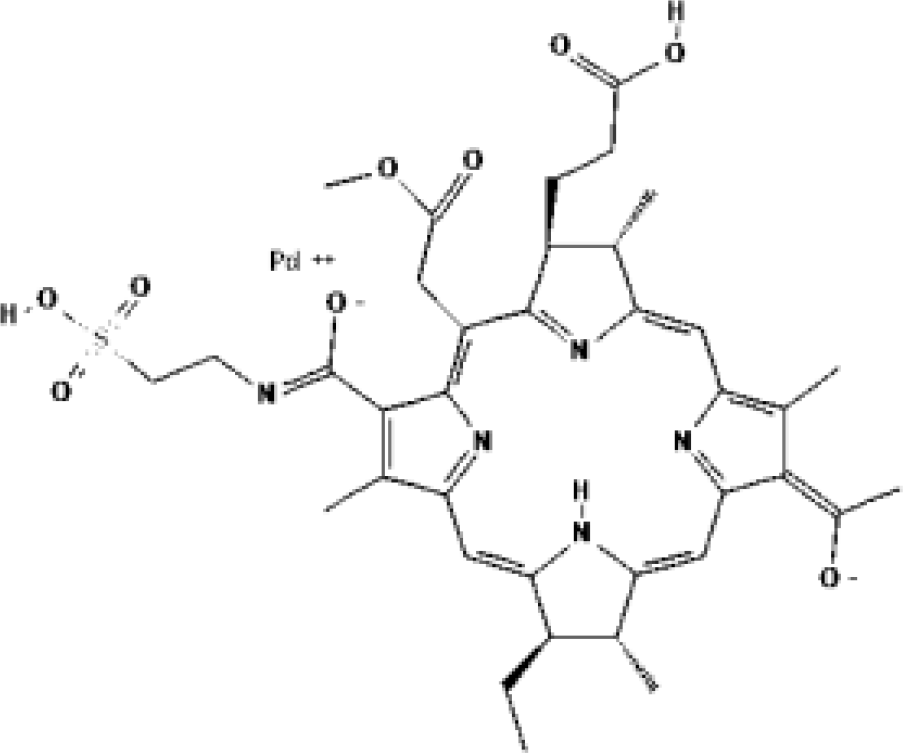		630 nm	Malignant mesothelioma, Lung cancer, Head and neck cancer
5-Aminolevulinic acid (5-ALA, Levulan^®^)	Porphyrin precursor	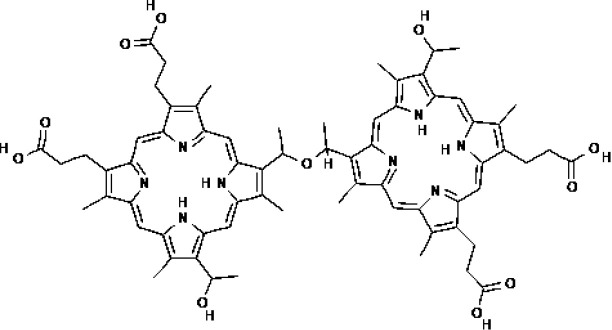		635 nm	Head and neck cancer guided resection, Brain cancer, Cutaneous T-cell lymphoma, Basal cell carcinoma, Breast cancer, Bladder cancer
Temoprfin (mTHPC, Foscan^®^)	Chlorin	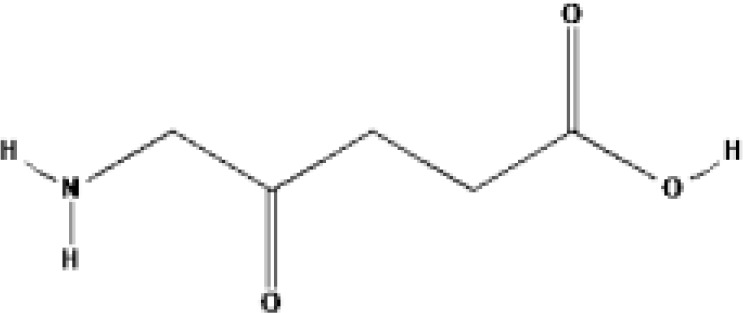		652 nm	Nasopharyngeal cxarcinoma, Head and neck cancer, Bile duct carcinoma
Verteporfin (BPD-MA, Visudyne^®^)	Chlorin	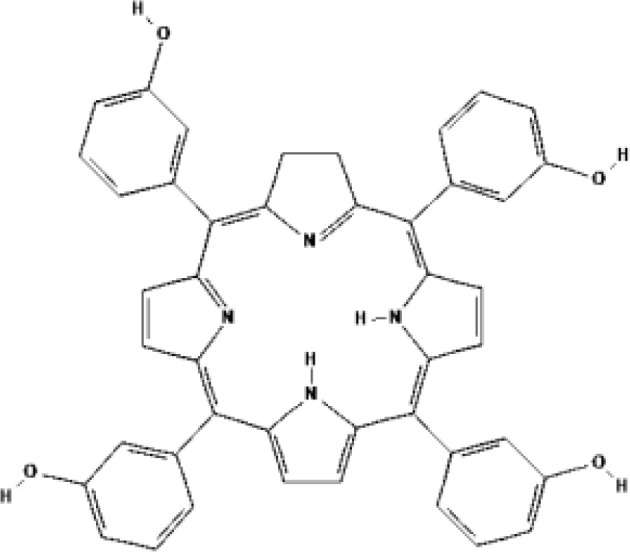		690 nm	Glioblastoma, Recurrent prostate cancer, Pancreatic carcinoma, Breast cancer, Solid tumor
Talaporfin (Npe6, Laserphyrin^®^)	Chlorin	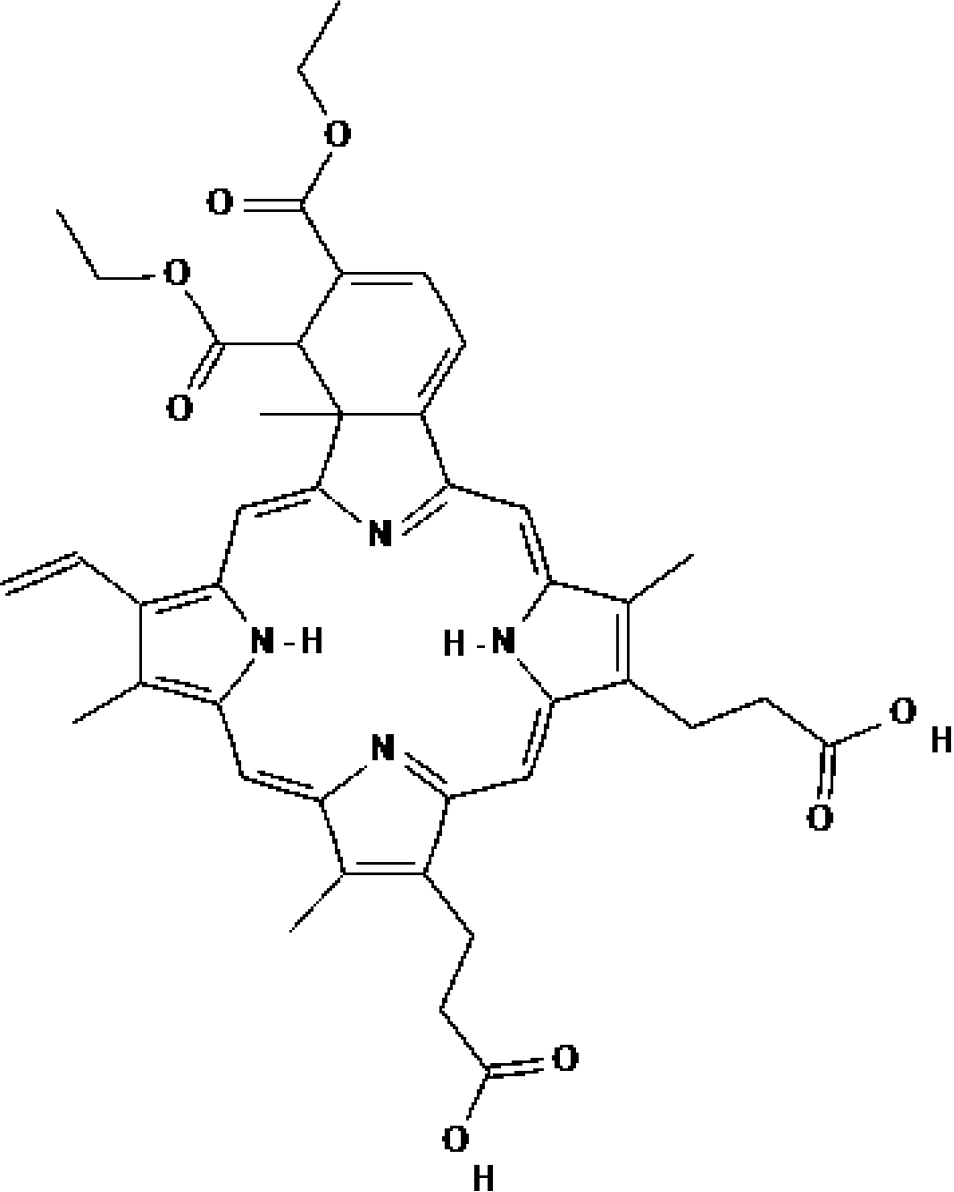		660 nm	Hepatocellular carcinoma, Liver neoplasms, Head and neck cancer
2-(1-Hexyloxyethyl)-2 devinyl pyropheophorbide-A (HPPH, Photochlor^®^)	Chlorin	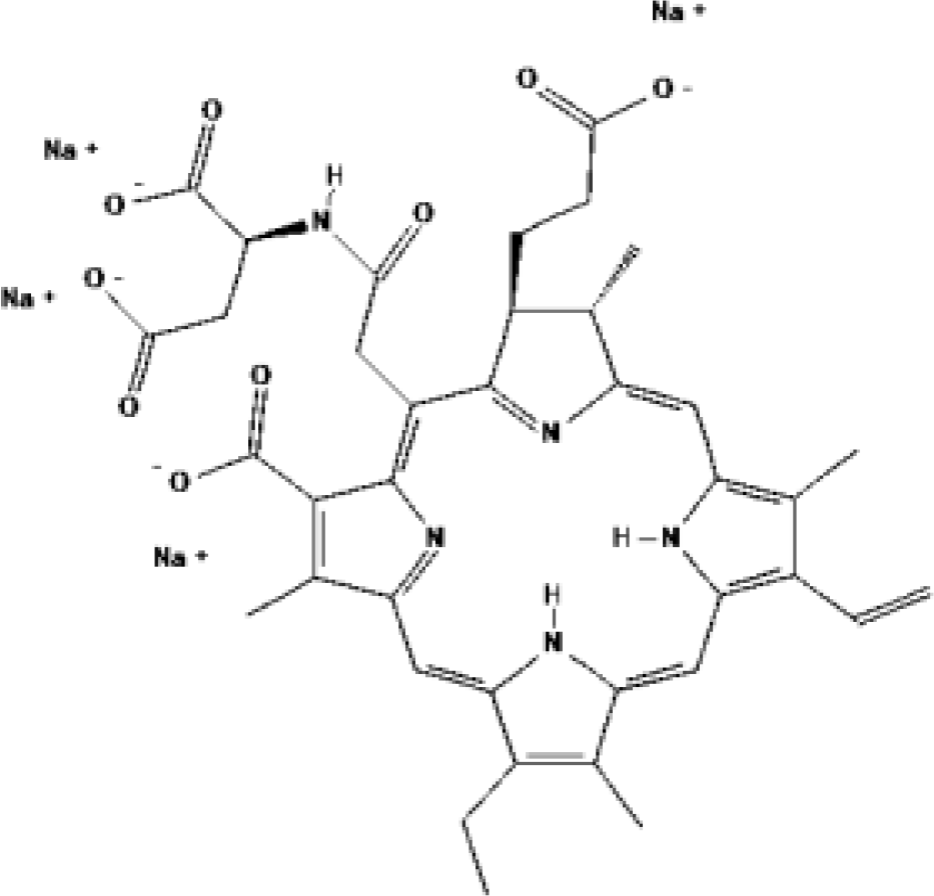		665 nm	Lung cancer, Head and neck cancer
Chlorine a6 (Photolon^®^)	Chlorin	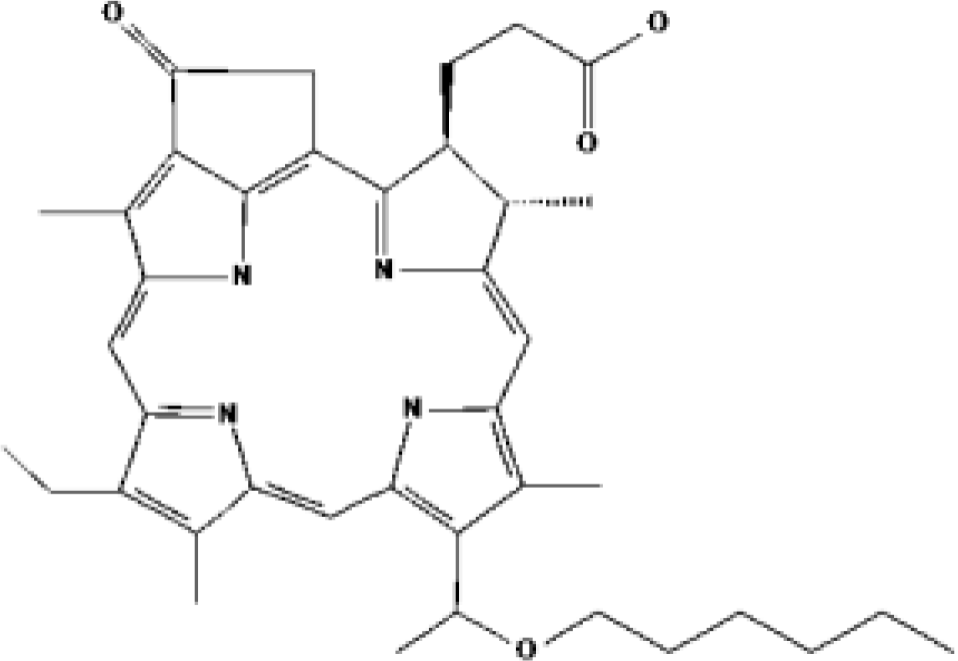		660 nm	Hilar cholangiocarcinoma
Silicon phthalocyanine (Pc4)	Phthalocyanine	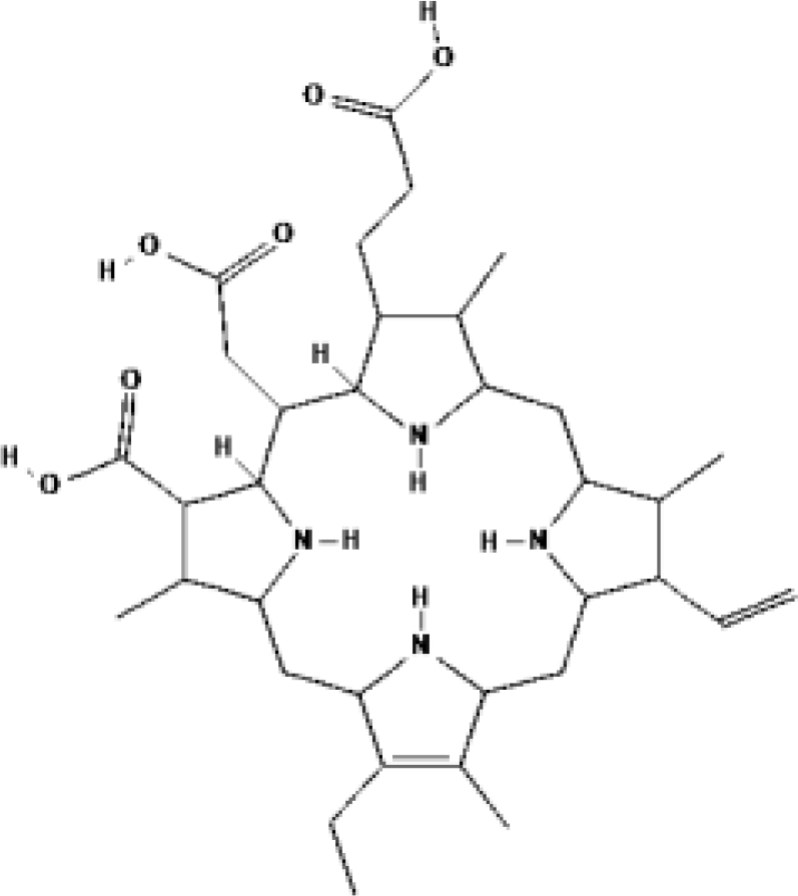		675 nm	Cutaneous T-cell lymphoma, Non-melanomatous skin cancer
Redaporfin (LUZ11, F_2_Bmet)	Bacteriochlorin	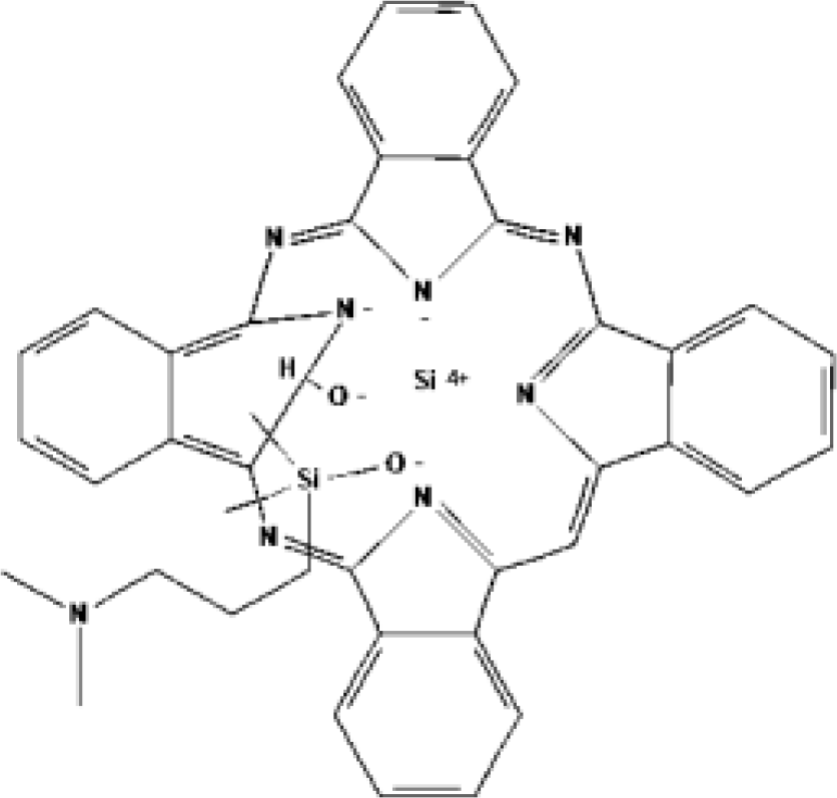		749 nm	Head and neck cancer
RM-1929	Cetuximab-IR700 conjugate	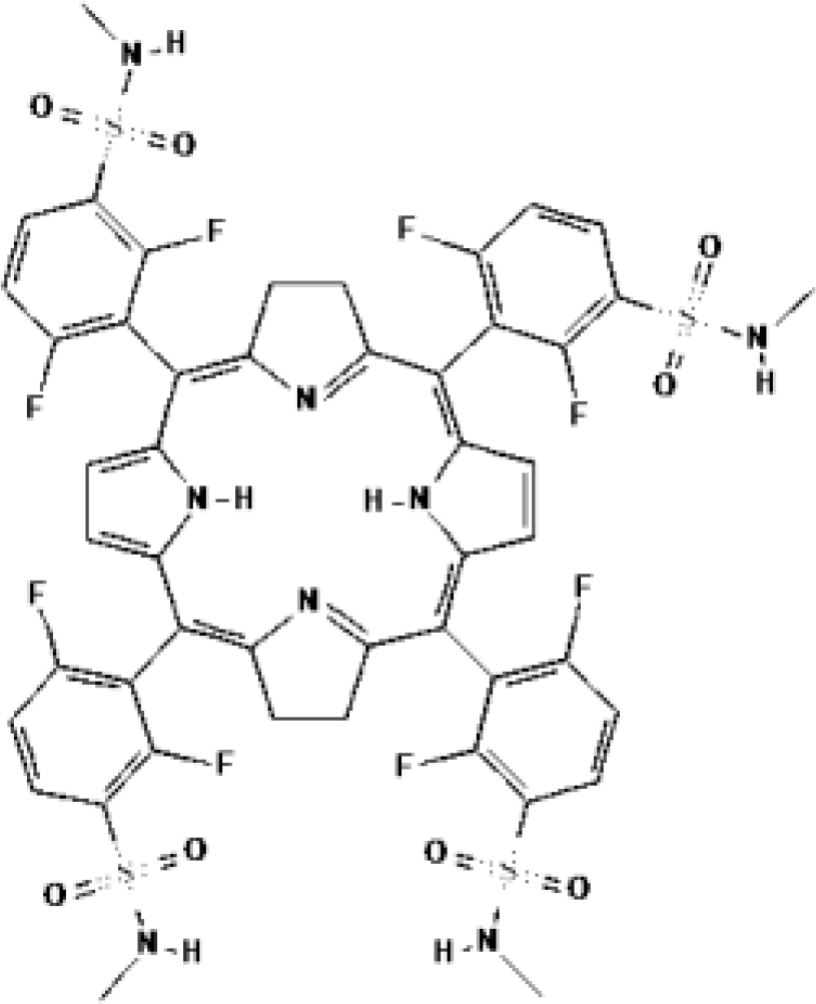		689 nm	Head and neck cancer

### Light delivery systems

The first light delivery devices for PDT were simple lamps, delivering white light (a broad spectrum of wavelengths). For the maximal photodynamic efficiency to eliminate tumors during PDT, PSs must be activated by light of a specific wavelength by producing the most ROS. In order to be easily guided into the body for tumor irradiation, the light intensity must be sufficient and efficiently transferred through the optical fiber. Semiconductor lasers, helium-neon lasers, light-emitting diode (LED) light sources, etc. are a few examples of the light source emitters (23).

**Figure 2:**
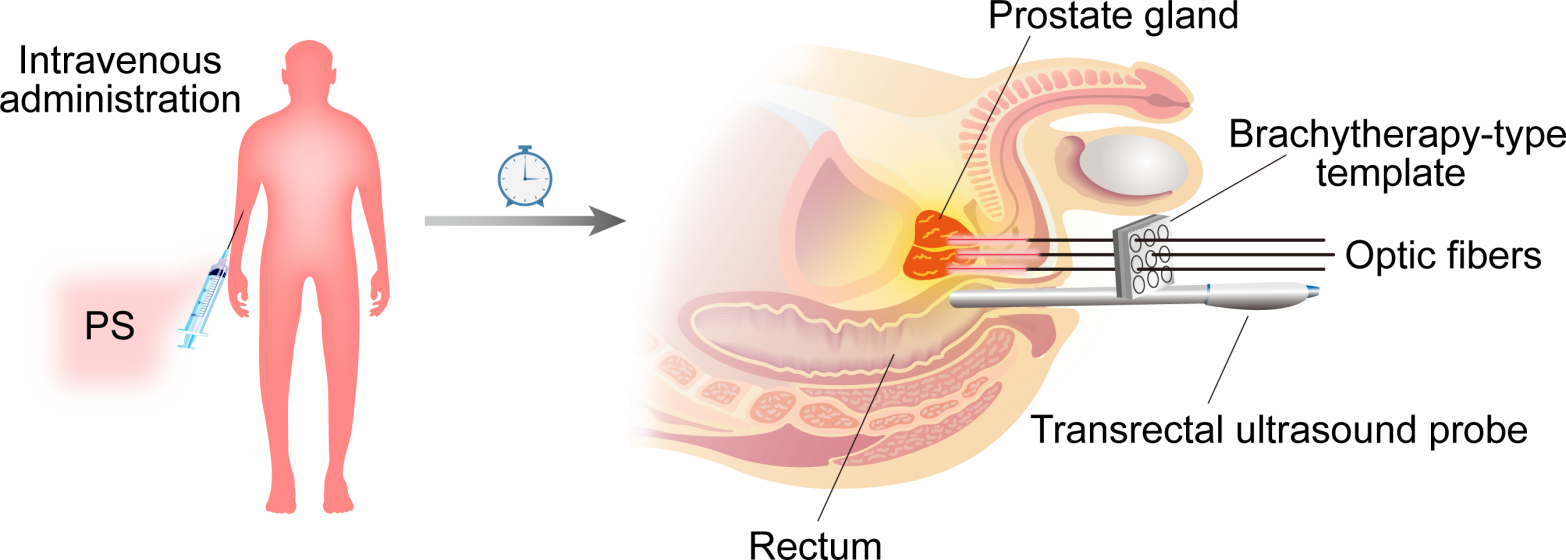
Mechanisms of PDT on tumors. Upon light activation, the ^1^PS is converted from a ground state (S_0_) to the excited singlet state (S_1_) as ^1^PS^•^. ^1^PS^•^ is excited to the triplet state (T_1_) as ^3^PS^•^ *via* intersystem crossing. Further, ^3^PS^•^ promotes the generation of ROS through two mechanisms: type I reactions involve the formation of ROS, such as peroxides (H_2_O_2_, ROOH), superoxide anion(O_2_^-•^), hydroxyl radical (HO^•^) and hydroxyl radicals (HOO^•^). Type II, the energy from ^3^PS^•^ is directly transferred to triplet state oxygen (^3^O_2_) to form singlet oxygen (^1^O_2_). Ultimately leading to cellular toxicity, recruitment and activation of immune cells and vascular damage.

The diode laser, which produces high-energy, single-wavelength light, is the most widely used PDT since it is practical, portable, cohesive, and monochromatic, and because its output power can be precisely controlled. It can be directly passed through optic fiber cables into hollow organs to irradiate tumors (24). For prostate cancer, the introduction of optical fibers was percutaneous intervention guided by an imaging system. Following a period of intravenous administration of PS, as shown in **Figure 2**, the patient is usually placed under general anesthesia in a lithotripsy position and a urethral Foley catheter is placed. As used in brachytherapy, a template grid is placed on the perineum to aid needle positioning. Catheter needles within optical fibers are positioned and guided to the prostate gland through a brachytherapy template in the perineum by transrectal ultrasound (TRUS) or MRI (25). A light detection probe is also placed in the rectum to ensure that the light dose is low enough to minimize energy transfer to areas outside the prostate. Additional laser radiation is applied to the tumor.

## Clinical studies of PDT for prostate cancer

For a long time, doctors have carried out numerous PDT clinical trials for prostate cancer. The different PS-mediated PDT are discussed in the sections below (**Table 3**).

**Table 3:** Clinical trials of PDT for prostate cancer.

PSs	Time (Y)	Clinical trial stage	Mean age (Y)	Median PSA (ng/ml) prior to PDT	Drug dose/Light dose/Drug-light interval	λ_max_	Fiber	Fiber insertion method	Ref.
**Hpd** **Photofrin**	1999	n.a.	n.a.	n.a.	1.5 mg kg^-1^/15 J cm^-1^/48-72 h 2.5 mg kg^-1^/15 J cm^-1^/48-72 h	628 nm	Spherical light diffuser	transurethral	(26)
**m-THPC**	2002	phase I	68 (58–77)	27.6 (11.8-37.3)	0.15 mg kg^-1^/20 J cm^-1^ or 50 J cm^-1^/3 d	652 nm	bare tip fiber and 1 treatment with cylindrical diffusers	perineal	(27)
	2006	phase I	66 (61-71)	5.25 (1.9-15)	0.15 mg kg^-1^/50-100 J cm^-1/^2, 3 and 5 d	652 nm	bare tip fiber and cylindrical diffusers	perineal	(28)
**Oral 5-ALA**	2003	phase I	68.6 (58-76)	7 (4.9-10.9)	20 mg kg^-1^/250 J cm^-1^/4 h	380-440 nm 633 nm	cylindrical diffusers	3 transurethral	(29)
	2009	phase II	n.a.	10 (2.3-120)	20 mg kg^-1^ (and ≦ 1.5 g/person)/3 h	380-420 nm	cylindrical diffusers	n.a.	(30)
**Motexafin Lutetium (MLu)**	2006	phase I	median age 69 (57-79)	6.4 (1.8-15.4)	0.5 mg kg^-1^/25 J cm^-1^/24 h 1 mg kg^-1^/25 J cm^-1^/24 and 6 h 2 mg kg^-1^/25, 50, 100 and 150 J cm^-1^/6 and 3h	732 nm	cylindrical diffusers	perineal	(31) (32)
**Padoporfin (Tookad^®^ WST09)**	2007	phase I	n.a.	< 20	0.1, 0.25, 0.5 and 1 mg kg^-1^/100 J cm^-1^/10 min 2 mg kg^-1^/230 and 360 J cm^-1^/6 min	763 nm	cylindrical diffusers	perineal	(33)
	2008	phase II	n.a.	n.a.	2 mg kg^-1^/>23 J cm^-2^/6 min	763 nm	cylindrical diffusers	perineal	(34)
**Padeliporfin (Tookad^®^ WST11)**	2013	phase II	62.7	mean PSA 6.38 (0.8-12.9)	4 or 6 mg kg^-1^/200 J cm^-1^/0 min 4 mg kg^-1^/200 and 300 J cm^-1^/0 min	753 nm	cylindrical diffusers	perineal	(35)
	2013	phase II	63 (55-75)	mean PSA 6.2 (1.3-9.8)	4 mg kg^-1^/200 J cm^-1^/0 min	753 nm	cylindrical diffusers	perineal	(36)
	2015	phase II	63.9	≤ 10	2, 4 and 6 mg kg^-1^/200 J cm^-1^/0 min	753 nm	cylindrical diffusers	perineal	(37)
	2016	phase I/II	61.6 (47-74)	≤ 10	4 mg kg^-1^/200 J cm^-1^/0 min	753 nm	cylindrical diffusers	perineal	(38)
	2017	phase III	64.2 (45-85)	mean PSA 6.2 (0.1-10)	4 mg kg^-1^/200 J cm^-1^/0 min	753 nm	cylindrical diffusers	perineal	(39) (40)
	2017	phase II	median age 63 (51-76)	6.1 (1.3-10)	4 mg kg^-1^/150 and 200 J cm^-1^/0 min	753 nm	cylindrical diffusers	perineal	(41)
	2018	phase II	65,3 ± 7,38	8,69 ± 5,68	4 mg kg^-1^/150 and 200 J cm^-1^/0 min	753 nm	cylindrical diffusers	perineal	(42)
	2019	phase II	62.6	< 10	4 mg kg^-1^/200 J cm^-1^/0 min	753 nm	cylindrical diffusers	perineal	(43)
	2022	real-world	63	< 10	4 mg kg^-1^/200 J cm^-1^/0 min	753 nm	cylindrical diffusers	perineal	(44)

n.a., not applicable.

### PDT with various PSs

The Lancet published the first clinical study on PDT for prostate cancer in 1990 (26). Windahl et al. performed transurethral PDT with spherical light diffuser after transurethral radical prostatectomy in 2 patients. One person was taken with HpD and another was Photofrin^®^. There were no complications and PSA dropped to 2.5 and 0.2 ng/ml, respectively after five months. Following a random biopsy, there were no histological evidence of tumor was discovered at three to six months. To minimize phototoxicity, patients were advised to avoid direct sunlight for 6 weeks following PDT and to wait 3 days between intravenous injection and irradiation.

Temoporfin (5,10,15,20-Tetra (m-hydroxyphenyl) chloride, m-THPC, Foscan^®^) is a second-generation PS, which was approved by EMA for advanced head and neck cancer. It can be activated at 652 nm with a light penetration depth of 1 cm (45). Many doctors tried to use it to treat prostate cancer. The first phase I study was reported by Nathan et al. in 2002 (27). Foscan^®^-PDT was given to 14 patients (median age 70 years) with local recurrence of prostate cancer after radiotherapy. The interval between intravenous injection and irradiation lasted 72 h, meaning the patients spent three days in a dark room. Then 13 of them were given high light dose (50 J) irradiation. At follow-up after PDT, PSA level decreased in 9 patients and no tumor was found in biopsies in 5 patients. CT and MRI showed that 91% necrosis of prostate cross section. 4 patients had stress incontinence, which were remission later. Sexual function of 4 patients was impaired. Unfortunately, PSA level of all patients eventually increased and they received ADT after 3 to 38 months. The researchers believed the reason may be that the irradiation did not cover the entire gland. In 2006, another phase I/II study of temoporfin-PDT was reported by doctors in London. Six patients (mean age 66 years, Gleason score 3 + 3 in all) with prostate cancer received focal or hemigland PDT treatments (28). Four of them had two treatments. The mean reduction of PSA value is 48.3% after 8 of 10 PDT treatments. Necrosis and fibrosis were found in treated areas by biopsies at 1-2 months. One patient developed mild stress and urge incontinence that resolved spontaneously within 4 months. All patients had residual cancer in at least one biopsy after PDT. 3 received external beam radiotherapy, 1 received brachytherapy, and 1 received cryotherapy. Only one remains well 6 years after PDT. This study pointed out that prolonged period of skin photosensitivity is a serious disadvantage of temoporfin. Both trails were limited to areas of cancer as detected by biopsy. With the accuracy of needle placement and improvements in PS and light dosimetry, the efficacy will be enhanced without affecting adjacent organs and normal tissues, such as the bladder, rectum, and neurovascular bundle.

5-Aminolevulinic acid (ALA) is the precursor of heme and PpIX. It can produce PpIX with strong photosensitivity through a series of enzymatic actions. PpIX can be activated at 420-640 nm. There are also two reports about ALA-PDT for prostate cancer. A few clinical studies in Germany reported the localization and efficacy of ALA induced PPIX in prostate cancer (29). 14 patients with prostate cancer (age 52-70 years, Gleason score 4-8) took oral ALA (20 mg/kg) and received RP 4 h later. Then surface of organ was investigated for PPIX fluorescence after violet light (380-440 nm), then frozen sections of the removed tissue were examined by fluorescence microscopy. The results showed that fluorescence was only found in cancer cells. Then 5 patients received PDT irradiation (633 nm). The PSA value reduced by 20% up to 70% after interstitial PDT 6 weeks later, then increased slowly one year later. No complications were reported. Adam et al. reported the feasibility of identifying positive surgical margins (PSM) by ALA in open retropubic or endoscopic extraperitoneal RP to improve surgical radicality (30). 39 patients with prostate cancer (Gleason score 6-10) took oral ALA (20 mg/kg but not ≤ 1.5 g/person) and received RP (24 for endoscopic extraperitoneal RP and 15 for open retroperitoneal RP). The results showed there are more false-negative cases but less false-positive in the open groups than in the endoscopic groups. The sensitivity of the endoscopic groups was much higher (75%) than that of open groups. The authors believed that ALA-PDD during RP might be a feasible and effective method. The significant advantage of ALA-PDD/PDT is the interval between drug with irradiation reduced to 4 h.

Motexafin Lutetium (MLu, Lutex^®^, Lutrin^®^), is a water-soluble pentadentate aromatic metallotexaphyrin, which absorbs strongly at 730-770 nm (46). And it was approved by FDA for breast cancer and malignant melanomas (47). Doctors in University of Pennsylvania School of Medicine have performed a series of MLu-PDT studies on prostate cancer. In 2006, Du et al. reported a phase I study about MLu-PDT for 16 patients (median age 69 years, Gleason score 6-9) with recurrent prostate cancer (31). Since the efficacy of PDT is closely related to the tumor hypoxic (48), in addition to parameters such as luminous flux, drug concentration, and drug-irradiation interval, the dose of blood flow oxygenation is measured for the first time. This study is also the first to assess PSA value from 1 day to several weeks after PDT. The results showed PSA increased significantly after PDT in a short time. The authors suggest that the transient increase in PSA may result from cellular damage induced by PDT, resulting in the release of PSA into the circulation. As in other clinical trials, PSA value of some patients dropped below baseline within 1-2 months after PDT, and then become rise again (32). This article also mentioned that PSA value may be related to photobleaching of MLu. They believed photobleaching of PS should be measured in PDT clinic trails (49).

Tookad^®^ are palladium-bacteriopheophorbide, bacteriophorbide derivatives of bacteriochlrorophyll. They can be activated by NIR light at 763 nm, which can penetrate tissue to a depth of 2 cm. Tookad^®^ are co-developed by biologists Yoram Salomon and plant photochemist Avigdor Scherz from the Weizmann Institute of Science in Israel. They are a kind of vascular-targeted PSs, which kill cancer cells by destroying tumor blood vessels. PDT mediated by Tookad^®^ is known as vascular-targeted photodynamic therapy (VTP). Padoporfin (WST09, Tookad^®^) is hydrophobic and Padeliporfin (WST11, Tookad^®^) is hydrophyllic. Trachtenberg et al. reported the first phase I/II a clinical trial of VTP in patients with recurrent prostate cancer following external beam radiotherapy (EBRT) (49). In this first trial, only two treatment fibers were placed in the prostate, with the primary goal to demonstrate safety (50). The first group of 24 patients (Gleason score > 6) received WST09 (0.1, 0.25, 0.5, 1 and 2 mg/kg) with the light dose at 100 J/cm. The other groups received WST09 dose of 2 mg/kg with the light dose at 230 J/cm or 360 J/cm. Next, they reported a phase II trial that 28 patients received WST09 with 2 mg/kg (34). The results showed the therapeutic effect is considerable with safe drug concentration and appropriate light intensity. Eight of 13 patients with D_90_ (the minimum light dose received by 90% of the prostate volume) > 23 J/cm^2^ had a complete response, with early MRI showing marked vascular loss of the prostate, and 6-month biopsy showing no residual cancer. No incontinence, impaired sexual function and rectal damage were reported. The VTP was subsequently introduced into Europe by Mark Emberton at the University College Hospital, London (35). 85 patients (Gleason score 3 + 3) with localized prostate cancer were given WST11-VTP. WST11 was water soluble, making it easier to utilize. The study included 2 parts. In the first part, patients with prostate size < 60 ml received 4 mg/kg WST11 and patients with prostate size ≥ 60 ml received 6 mg/kg WST11. The light dose was 200 J/cm. In the other part, patients were assigned to received 200 or 300 J/cm light based on predefined criteria. 6-month follow-up data showed that 4 mg/kg WST11 and 200 J/cm light were optimal, resulting in more than 80% of patients having negative biopsy. Another phase II trial in France reported that 56 patients (Mean age 63 years, Gleason score ≤ 3 + 3) received VTP. The mean PSA value after treatment for 6 months was 3.7 ng/ml and histopathological data confirmed that no residual tumor in the targeted area (36). To further determine the WST11 concentration and light dose, 40 patients (Gleason score ≤ 3 + 3) with low-risk prostate cancer were received 2, 4 or 6 mg/kg WST11 and 200 J/cm (37). The results indicated that 4 mg/kg WST11 and 200 J/cm light at 753 nm was the best parameters. 95% of treatments were effective of 12 men used these parameters and 83% had negative biopsy at 6 months (51). This therapeutic effect was also validated in a clinical study in the United States (38). VTP treatment with WST11 at 4 mg/kg and 200 J/cm was well tolerated (52). The most famous study was the European phase III randomized controlled trial comparing WST11-VTP with active surveillance, over 400 patients in 10 European countries were participated in the study (39). 206 patients assigned to VTP group and 207 patients assigned to active surveillance group. After a 2 years follow-up, 28% of patients in the VTP group versus 58% of patients in the active surveillance group experienced disease progression. 49% of patients in the VTP group versus 14% of patients in the active surveillance group had negative biopsy at 2 years. The serious adverse event in the VTP group was retention of urine, which resolved within 2 months. 266 patients were followed for 4 or more years, including 147 in the VTP group and 119 in the active surveillance group. Compared with active surveillance, VTP had a lower rate of conversion to RP at 4 years. But there was little difference in metastasis and overall survival rate between two groups (40). Because of the results of PCM301, the EMA approved WST11 for patients with previously untreated, unilateral, low-risk prostate cancer. There are also a number of clinical trials investigating the effectiveness of WST11 (41). A Latin American trial, PCM304, evaluated the efficacy of VTP hemiablation in men with low- and intermediate-risk prostate cancer, including those with bilateral Gleason 3 + 3 and 3 + 4 disease. 60 (74%) patients had negative biopsies at 12 months after VTP (42). In 2019, another phase II trial was to assess the medium-term tumor control in 68 patients with VTP in a 3.5-year follow-up (43). The results showed similar results to earlier phase II studies. 25% of patients had positive biopsies at 3.5 years. In addition, 11% of patients increased their Gleason score by 1 point. However, Eggener et al. reported that the rate of histopathological upgrade of patients with active surveillance was just 5.7% (53). The need for VTP should be carefully decided in patients with low or very low risk. This may be the reason why Tookad^®^-VTP was ultimately not approved by the FDA for prostate cancer despite its many advantages, such as no obvious side effects, almost no drug-light interval, metabolized easily by body. Extremely few people feel strong feelings of regret 12 months after VTP (54). Recently, a Germany real-world study reported patients initial experience of patients treated with VTP for unilateral low-risk prostate cancer (44). They compared short term functional and oncological outcomes with those of a consecutive cohort of patients undergoing RP. The results showed both low- and intermediate-risk prostate cancer was detected in 27% of patients by biopsy at 12 and 24 months after VTP. None of the RP patients had a prostate cancer recurrence. Erectile function retention: VTP (71%) and RP (30%), almost all patients with RP have urinary incontinence, 96% used one and 4% used two or more pads per day. This research suggests that VTP for low-risk prostate cancer has a lower complication than RP. However, recurrence and progression after VTP are common. Therefore, a rigorous surveillance strategy is required.

Notably, salvage radical prostatectomy after VTP appears to be feasible as it is not thermal ablation (55). A recent retrospective series reported 45 patients who received salvage RP with recurrent prostate cancer after VTP with a median operative time of 180 minutes and a median blood loss of 200 ml. Surgeons said RP was “easy” in 29 (69%) patients and “difficult” in 13 (31%). Surgeons reported that lateral fibrosis made nerve bundle dissection difficult on the VTP treated side, but no rectal injury or anastomotic stricture occurred. The nerve-sparing technique was used in 14 (33%) patients, with a positive surgical margin rate of 31%. The complication rate was 12% (2 patients were grade 1, 2 were grade 2, and 1 was grade 3) (56). The study suggests that salvage RP after VTP is feasible and safe without significant issues.

### Light delivery devices

The light delivery system consisting of a diode laser and optical fibers is essential to deliver the appropriate wavelength of light with minimal loss for PS activation. Early clinical trials for prostate cancer used “bare-tip” optical fibers, with light coming out at the end, like a flashlight (26–28). The unit of light dose is usually J/cm^2^ because light is distributed in all directions from the end of the fiber. But “bare-tip” optical fibers are more suitable for treating superficial lesions, either on the skin or within a hollow organ accessible by endoscope. In solid organs such as the prostate, cylindrical diffusers are more commonly used to allow light delivery along a given length at the distal end of the fiber. The unit of light dose is J/cm. It is worth mentioning that the light dose in PDT is not intended to produce a thermal effect, but only to activate the PS. Therefore, its light dose is much lower than that used for other prostate applications, such as YAG laser for holmium laser enucleation of the prostate (HoLEP), potassium titanate (KTP) laser for photoselective vaporization of the prostate (PVP). Only five patients have received transurethral PDT to date (26, 29). Although no complications such as incontinence or dysuria have been reported, other studies have suggested that transurethral PDT can induce urethral strictures (50). Especially for the larger prostate, the optical fiber is inserted through the perineum. Early clinical work employed a freehand transperineal placement of optical fibers (27–29). With the development of template-mapping biopsy and brachytherapy, the approach is now to use a stepper device to secure transrectal ultrasound (TRUS) and transperineal templates to assist fiber placement.

### Monitor system and software

Many studies have shown that light, PS, oxygen, and tissue optical properties within the prostate intra-patient and inter-patient vary widely (57). The PS concentration, tissue oxygenation and light flux are monitored during PDT, enabling real-time, patient-specific, and optimized light delivery. The dose of most PSs can only be monitored and cannot be adjusted during treatment, because drug delivery is usually hours to days before irradiation (except for Tookad^®^, which is injected immediately before irradiation). Additionally, tissue oxygen content can be monitored but not adjusted during treatment. Therefore, the only parameter that can really be adjusted is the light dose. PS monitoring is mainly achieved through fluorescence spectroscopy-based techniques (58). Tookad^®^ has negligible fluorescence due to its high triplet state quantum yield. Therefore, diffuse optical transmittance or reflectance techniques must be used for PS monitor. The near-infrared diffuse optical instrument is used to measure hemodynamic responses to PDT (59). The system combines diffuse reflectance spectroscopy (DRS), which measures blood oxygen content, with diffuse correlation spectroscopy, which measures tissue blood flow. Additional spectral-based measurements of blood oxygen saturation changes and PS dose during PDT are under investigation. Light dose monitoring is achieved by inserting optical detector fibers in the prostate, rectum and urethra, connected to a continuously recording light dosimeter.

In current PDT clinical applications, a combination of pre-treatment planning and online monitoring is used to develop individual treatment plans, determine the size and shape of the prostate on MR or ultrasound imaging, and estimate the optimal number, length, location and power of fibers from average tissue optical properties. Both the University Health Network and Steba Biotech company have developed this dosimetric planning platform software. The software calculates the PDT dose and necrosis boundaries for multiple delivery fibers using finite element solution of the diffusion approximation of the radiative transport equation, and then uses online measurement of fluence rate at the prostate boundary and/or tissue optical properties to adjust the light intensity. TOOGUIDE TRUS^®^ is a software guidance system that is designed to provide VTP therapy. It uses TRUS images to define the number, position, and illumination length of the fibers in order to maximize the dose of light to the target while sparing the surrounding tissues, such as the rectal wall, prostate apex, and urethra.

### Ongoing clinical studies

According to information from ClinicalTrials.gov, there are two PDT clinical trials for prostate cancer currently in progress, including drugs and devices. one single-center single-arm open-label, phase IIb study (NCT03315754/PCM204) at Memorial Sloan Kettering Cancer Center is currently investigating the efficacy, safety, and quality of life in patients with intermediate-risk prostate cancer after VTP. The study includes men with a histological diagnosis of Gleason 3 + 4 on one half of the prostate in no more than 2 sextants of the prostate gland and not present in more than 50% of any one core; cT2a-N0/Nx-M0/Mx; Prostate volume ≥ 25 ml and ≤ 70 ml; PSA ≤10 ng/ml. Tookad^®^ Soluble VTP treatment consist of the combination of 10 minute IV infusion of Tookad^®^ Soluble at the dose of 4 mg/kg, followed by the illumination of the zone to be treated with a 753 nm laser light delivered through transperineal interstitial optical fibers at a power of 150 mW/cm and light energy of 200 J/cm applied over 22 minutes and 15 seconds. After VTP hemiablation, patients will be followed for 5 years (60 months) with clinical evaluation, questionnaires on QOL, erectile and urinary functions, PSA testing, and prostate biopsy at 3, 12, 24, 36, 48 and 60 months. The primary objective is to evaluate for Gleason grade 4 or 5 prostate cancer at 12 months by biopsy. The results of this study will provide meaningful information on medium-term oncologic outcomes in men with intermediate- risk prostate cancer treated with VTP.

Another Open-label Clinical Study (NCT03067051) at Princess Margaret Cancer Centre and University College London is studying the safety and adequacy of effectiveness of the SpectraCure P18 system (Interstitial Multiple Diode Lasers and IDOSE^®^ software) and Verteporfin for Injection (VFI) for patients with local recurrence after radiotherapy. The study includes men with expected survival ≥ 8 months; Eastern Cooperative Oncology Group (ECOG) performance status of 0 or 1; prostate volume ≤ 50 cm^3^; sufficient bone marrow reserve (granulocyte count≥1500/mm^3^, platelet count ≥ 100,000/mm^3^), adequate renal and hepatic function (creatinine ≤ 1.5 mg/dl, a total bilirubin ≤ 1.5 mg/dl, serum glutamate-oxaloacetate transaminase (SGOT) ≤ 3 times the upper limit of normal, alanine transaminase (ALT) ≤ 3 times the upper limit of normal); Patients with locally advanced (AJCC 7th edition T3/T4) or metastatic disease, Gleason score≥8 at initial diagnosis, and seed implantation brachytherapy before are excluded. The study design for accelerated titration of light dose and VFI. Potential damage to the periprostatic tissue including the rectal wall will be evaluated by contrast-enhanced and not-contrast enhanced MRI at 5-9 days following PDT. Effectivity will be evaluated by MRI to determine the extent of necrosis in the prostate within 1 week. Performance of the SpectraCure P18 system will be evaluated by light dose-volume histograms for the light dose coverage at 12 months.

## Preclinical researches of PDT for prostate cancer

As PDT has been approved in more and more countries, it has become a new routine treatment option in more and more hospitals, and preclinical research is also in progress. Here, preclinical studies of PDT for prostate cancer from 2009 to the present are discussed here to show what are currently available to overcome problems in recent clinical trials.

### Strategies for targeting improvement

In order to reduce the PS toxicity in normal tissues and enhance tumor killing effect, many studies have been carried out to improve the tumor targeting of PS as shown in **Table 4**, including passive targeting and active targeting. Passive targeting is generally delivered through nanoparticle systems, which contains liposomes, micelles, polydopamine, inorganic nanomaterials, etc (87). The mechanism is mainly to enhanced permeability and retention (EPR) effect by adjusting the size of nanoparticles and modifying the surface chemistry. In solid tumor tissue, there are abundant blood vessels, wide vascular wall space, poor structural integrity, and lack of lymphatic return, resulting in selective high permeability and retention of macromolecules and nanoparticles (88). Duchi et al. developed 80 nm shell-core fluorescent nanoparticles (FNP), the shell was made of polymethylmethacrylate, and loaded with the PS tetrasulfonated aluminum phthalocyanine (Ptl). Ptl@FNP were concentrated on prostate cancer cells through EPR effect (60). However, the EPR effect is controversial. In 2020, a study was reported by Chan et al. that up to 97% of nanoparticles enter the tumor through active processes of endothelial cells (89). The results may influence the development of nanoparticles of PS in the future.

**Table 4:** Different preclinical studies of PDT for prostate cancer.

Molecular Conjugates PS/NPs	Target moiety	λ_max_	Cell model	Light dose *in vitro*	Animal model	Light dose *in vivo*	Ref.
**Ptl@Fluorescent NPs**	EPR	680 nm	PC3	263 J cm^-2^/876.6 mW cm^-2^ and 1581 J cm^-2^/878.3 mW cm^-2^	6-week-old SCID mice	8.04 J cm^-2^/26.8 mW cm^-2^	(60)
**PSMA-Ppa**	PSMA	600-800 nm	LNCaP	7.5 J cm^-2^/25 mW cm^-2^	n.a.	n.a.	(61)
**PSMA-IR700**	PSMA	690 ± 20 nm	PSMA^+^ PC3PIP and PSMA^-^ PC3flu	2 J cm^-2^/n.a.	6- to 8-week-old male NOD-SCID mice	100 J cm^-2^/n.a.	(62)
**PSMA-1-Pc413 PSMA-1-IR700**	PSMA	672 nm 690 nm	PSMA^+^ PC3pip and PSMA^-^ PC3flu	0.5 J cm^-2^/8.3 mW cm^-2^	6- to 8-week-old male athymic nude mice	150 J cm^-2^/33.3 mW cm^-2^ and 50 J cm^-2^/31.8 mW cm^-2^	(63)
**AuNP-5kPEG-PSMA-1-Pc4**	PSMA	672 nm	PSMA^+^ PC3pip and PSMA^-^ PC3flu	0.1, 0.5 and 1 J cm^-2^/1-5 mW cm^-2^	6- to 8-week-old male athymic nude mice	150 and 300 J cm^-2^/0.1 W cm^-2^	(64)
**AuNP-Pc158**	PSMA	670 nm	PSMA^+^ PC3pip and PSMA^-^ PC3flu	1 and 150 J cm^-2^/n.a.	6- to 8-week-old male athymic nude mice	150 J cm^-2^/n.a.	(65)
**^64^Cu-LC-Pyro**	PSMA	671 nm	PSMA^+^ PC3pip and PSMA^-^ PC3flu	0.5, 1, 2, 3 and 5 J cm^-2^/n.a.	Athymic male nude mice	100 J cm^-2^/55 mW cm^-2^	(66)
**BChl-LC-PSMA**	PSMA	750 nm	PSMA^+^ PC3pip and PSMA^-^ PC3flu	1, 2.5, 5, 10 and 15 J cm^-2^/50 mW cm^-2^	6- to 8-week-old male athymic nude mice	125 J cm^-2^/70 mW cm^-2^	(67)
**RGD-4R-MPD/TTB**	integrin α_ν_β_3_	730 nm	PC3	n.a./200 mW cm^-2^	5-week-old male BALB/c nude mice	n.a./200 mW cm^-2^	(68)
**c(RGDyK)-Phthalocyanine**	integrin α_ν_β_3_	660 nm	DU145	12 J cm^-2^/10 mW cm^-2^	n.a.	n.a.	(69)
**c(RGDyK)-SOC-UCNP-ZnPc**	integrin α_ν_β_3_	980 nm 660 nm	PC3	n.a./10, 22, 34, 49 and 65 mW cm^-2^	Athymic nude mice	n.a./34 mW cm^-2^	(70)
**PpⅨ-PA**	PA	630 nm	PC3, DU145 and LNCaP	75 J cm^-2^/n.a.	5-week-old female BALB/c nu/nu mice	200 J cm^-2^/n.a.	(71)
**CoFe_2_O_4_-HP-FA**	FR	515 nm	PC3	3.06, 6.12, 9.18 and 18.36 J cm^-2^/n.a.	n.a.	n.a.	(72)
**Fe_3_O_4_-Ce6-FA**	FR	660 nm	PC3	36 J cm^-2^/20 mW cm^-2^	n.a.	n.a.	(73)
**MSN-M6C-PS**	M6PR	650 nm	LNCaP and DU145	6.5 J cm^-2^/n.a.	n.a.	n.a.	(74)
**MSN-M6C-Man**	M6PR	650 nm	LNCaP	11.25 J cm^-2^/3 mW cm^-2^	n.a.	n.a.	(75)
**Laserphyrin-HVJ-E**	HN	664 nm	castration-resistant PC3	99 mJ cm^-2^/150 mW cm^-2^	n.a.	n.a.	(76)
**ClAlPc-NC** **ClAlPc-NE**	EPR	650 nm	LNCaP	0.5, 4 and 7 J cm^-2^/20 92 mW cm^-2^	n.a.	n.a.	(77)
**Iridium biscyclometallated Ir (III) complexes**	mitochondria	450 nm	PC3	24.1 J cm^−2^/6.7 mW cm^−2^	n.a.	n.a.	(78)
**fluorinated porphyrinoids@PVP**	mitochondria	622 nm	PC3	10.6 J cm ^-2^/17.6 mW cm^-2^	n.a.	n.a.	(79)
**MC540/ZnPc-UCNP@Au**	EPR	980 nm	PC3	n.a./0.4, 0.6, 0.8, 1, and 1.2 W cm^-2^	n.a.	n.a.	(80)
**YPMS@PpⅨ@FA**	FR	UV_365 nm_	Tramp-C1 and Tramp-C2	0.1, 0.5, 1, 2 and 3 J cm^-2^/n.a. and 125, 250 and 500 mJ cm^-2^/n.a.	8-week-old male CD-1 mice	n.a.	(81)
**PEG-PLGA-LaF_3_:Ce^3+^/PpⅨ**	EPR	UV_403 nm_ and X-rays	PC3 and DU145	10 J cm^-2^ 5, 8 and 10 Gy	6- to 8-week-old C57bl/6 black mice	n.a.	(82)
**FA-PpⅨ-AG**	FR	X-rays	PC3 and PNT1A	1, 2, 4, 6 and 7 Gy	n.a.	n.a.	(83)
**OH-, Br- and Cl- Coelenterazine**	n.a.	chemiluminescence	PC3	n.a.	n.a.	n.a.	(84)
**SPION/Ce6/Oxygen-loaded polymer bubbles**	n.a.	660 nm	Tramp-C1	n.a./50 mW cm^-2^	6- to 8-week-old C57BL/6J Narl male mice	n.a./100 mW cm^-2^	(85)
**ICG/AIBI/17-AAG/HA**	CD44	808 nm	LNCaP	n.a./0.5, 1 and 2 W cm^-2^	n.a.	n.a.	(86)

NPs, Nanoparticles; Ptl, Tetrasulfonated aluminum phthalocyanine; Ppa, Pyropheophorbide-A; PSMA-1: Glu-CO-Glu′-Amc-Ahx-Glu-Glu-Glu-Lys-NH_2_; LC-Pyro: Long-circulating Pyropheophorbide; BChl, Bacteriochlorophyll; PA, Polyamine; MSNs, Mesoporous silica nanoparticles; M6C, Mannose 6-Carboxylate; M6C-Man, dimannoside-carboxylate; HVJ-E, Hemagglutinating virus of Japan envelope; HN, hemagglutinin-neuraminidase; NE, Nanoemulsions; NC, Nanocapsules; HP, Hematoporphyrin; UCNP, Upconversion nanoparticles; YPMS, Y_2.99_Pr_0.01_A_l5_O_12_ afterglow: AG, Sr_2_MgSi_2_O_7_:Eu^2+^, Dy^3+^ afterglow; CLa: ICG: indocyanine green; AIBI: (2,20-azobis[2-(2-imid- azolinI-2-yl) propane] dihydrochloride; 17-AAG, heat shock protein 90 inhibitor geldanamycin; HA, Hyaluronic acid.n.a., not applicable.

Active targeting to improve tumor selectivity typically through binding or adhering to specific receptors overexpressed on cancer cell surface, including proteins (e.g. antibodies), peptides (e.g. arginine-glycine-aspartate peptide and epidermal growth factor), aptamers, vitamins (e.g. folic acid and biotin) (90) and carbohydrates (91). Compared to antibody or antibody fragments, peptides and aptamers are rapidly cleared from the body due to their small size. Prostate-specific membrane antigen (PSMA) is a unique transmembrane glycoprotease on the cell surface with carboxypeptidase and folate hydrolase activities (92). It was initially discovered in the androgen-dependent LNCaP human prostate cancer cell line (93). The expression level of PSMA on prostate cancer cells is 100 times more than that on normal cells (94). And its expression level increases as the cancer progresses, making it a highly specific and sensitive marker to target for therapy (95). Most of the peptides utilized the PSMA binding moiety Lys-Glu urea to conjugate PS (96, 97). The first study was reported by Liu et al. in 2009 (98). They designed a PSMA inhibitor conjugate with PS pyropheophorbide-a (Ppa-CTT-54) for targeting prostate cancer. After PDT with Ppa-CTT-54, the apoptosis signaling pathway was activated, cytoskeletal was destroyed, leading to apoptosis and necrosis (61). Chen et al. reported another PSMA-targeted Lys-Glu urea based PS IRDye700DX (IR700) for prostate cancer (62), and evaluated its PDT efficacy for prostate cancer (99). IR700 is a NIR silica-phthalocyanine hydrophilic dye with photosensitivity. Wang et al. reported a new PSMA peptide-based ligand (PSMA-1) binding Glu-Glu urea moiety (63). PSMA-1 was utilized to conjugate PSs, Pc413 and IR700. Pc413 is a kind of second generation phthalocyanine dye Pc4. The results showed two PSMA-1-PDT conjugates can effectively inhibit PSMA^+^ PC3 cells progression (100). Mangadlao et al. published gold nanoparticles (AuNPs) based on PSMA-1 loaded with Pc4 was synthesized. The theranostic agent also exhibits promising targeting properties and phototoxicity of prostate cancer (64). In 2020, this team reported a multiple targeting nanoparticle delivery system. The core was AuNPs, which was conjugated to PSMA-1 and silicon phthalocyanine (Pc158) by a cathepsin-cleavable linker. The nanoparticles were endocytosed by prostate cancer cells based on PSMA targeting. Then due to a large amount of cathepsins in the intracellular lysosomal vesicles, the liker was cleavage and Pc158 was released over time (65). However, this rapid metabolism of small molecule peptides often results in insufficient tumor accumulation, requiring repeated administration of PS. To prolong tumor accumulation, Zheng et al. reported a 9-amino-acid D-peptide linker to prolonged plasma circulation. PS was conjugated of targeting ligands *via* the liker. Both PSs (Ppa and bacteriochlorophyll) conjugates showed good tumor tissue accumulation duration and PDT activity (66, 67).

The integrin α_ν_β_3_ is overexpressed on tumor vascular endothelial cells (101). The arginine-glycine-aspartate (RGD)-containing peptide ligand that exhibits strong binding affinity and selectivity for integrin α_ν_β_3_ (102). Dai et al. synthesized NPs combined RGD-4R (a modular peptide) with TTB (a fluorescein with aggregation-induced emission) for image-guided PDT treatment (68). Luan et al. synthesized a cyclic RGD peptide (c(RGDyK)) moiety conjugated with an unsymmetrical phthalocyanine. The results showed this phthalocyanines conjugate PS was highly enriched in α_v_β_3_-positive DU145 prostate cancer cells (69). Recently, a mechanistic study of phthalocyanines suggests that phthalocyanine derivatives with imidazole groups can be effectively used against prostate cancer while differentially effecting metastasis, angiogenesis, cell cycle, apoptosis and immune system cells’ activities (103). Another c(RGDyK) conjugate PS ZnPc platform (R-SUZn) was reported. Hydrophilic chitosan was used for ZnPc delivery and UCNPs were used to convert NIR light to visible light to increase illumination depth. To simulate deep tumors, 1 cm of pork tissue was overlaid on a subcutaneous prostate cancer tumor. The results showed enhanced photodynamic effects of this nanoparticle in the deep tumor (70). It has also been reported to target PDT in prostate cancer by conjugating polyamines (PA) to PpIX (71). PA was a class of aliphatic nitrogenous bases that was highly expressed in tumors. In addition, tumor cells have an up-regulated polyamine transport system (PTS) for the uptake of exogenous polyamines (104). Folic acid (FA) has been extensively studied for the targeted delivery of nanomedicines by recognizing the overexpressed folate receptor-α (FR-α) in many cancers (105). Choi et al. synthesized two kinds of FA modified nanoparticles for PDT to treat prostate cancer, HPs-conjugated multifunctional magnetic nanoparticles (CoFe_2_O_4_-HPs-FAs) (72) and Ce6-conjugated multifunctional magnetic nanoparticles (Fe_3_O_4_-Ce6-FAs) (73). The results indicated that Ce6-conjugate had better anticancer activity than HPs-conjugate after PDT. This may be related to the high yield of _1_O^2^ in Ce6. However, Wang et al. suggested that FA functionalization on the nanoparticle surface did not lead to more nanoparticle enrichment in FR-α-overexpressing tumors, but rather to nanoparticle capture by macrophages in tumors, liver, and spleen, which deprived FR of the ability to recognize and accelerate complement activation *in vivo* (106). This study may have challenges for the development of FA-targeted PSs. Cation-independent mannose 6-phosphate receptor (CI-M6PR), which is overexpressed in 84% of prostate cancers, has attracted the attention and appears to be a target for PDT in prostate cancer. One of the roles of CI-M6PR is endocytosis of proteins with mannose 6-phosphate (M6P) ligands. Bouffard et al. reported a new ligand with a better affinity for CI-M6PR, dimannoside-carboxylate (M6C-Man) (74). They synthesized the mesoporous silica nanoparticles (MSNs) loaded M6C-Man and monosaccharide carboxylate (M6C) for PDT in prostate cancer (75). The results showed M6C-Man nanoparticles were absorbed more rapidly by cells than monosaccharide carboxylate (M6C) nanoparticles, which may be related to the stronger activity of dimannoside derivative than the monosaccharide. The clinical challenges of treating castration-resistant prostate cancer (CRPC) are still enormous. PDT may become a new option for patients who are already hormone-resistant but have not yet metastasized. Scientists in Japan have tried to use Hemagglutinating virus of Japan envelope (HVJ-E), which is specific for prostate cancer, as a PS carrier for PDT in CRPC (76). Hemagglutinating virus of Japan envelope (HVJ-E) is oncolytic virus, which can induce CRPC PC3 and DU145 cell membrane fusion (107). This makes the PS conjugate (Laserphyrin^®^-HVJ-E) not only targeted but also cytotoxic of HVJ-E (108). Mitochondria are organelles closely related to ROS generation, so targeting mitochondria is a very effective strategy. Iridium biscyclometallated Ir (III) complexes have been synthesized to target mitochondria. Photoactivation of the complex induces mitochondrial membrane depolarization and DNA damage, which triggers apoptosis (78, 109). Mesquita MQ et al. reported a three fluorinated porphyrinoid derivatives and entrapped them into polyvinylpyrrolidone (PVP). Two of them were localized in mitochondria and further killed PC3 cells (79).

### Light strategies to overcome depth limitations

A good light source of PDT should have the following characteristics: a spectral range consistent with the peak absorption wavelength of the applied PS and sufficient tissue penetration depth. Most peak absorption wavelength of PSs was in the visible range. Due to the poor tissue penetration depth of excitation lights, the penetration depth of green or blue light is only 2 mm, which is not enough for deep tumors. Therefore, there is an urgent need to find out some new strategies to produce ROS under external stimulation with high penetrability (110). Several third-generation of PSs can respond to external excitations such as NIR light, ultrasound, X-ray, chemiluminescent, bioluminescent sources, Cherenkov radiation, and implants (111). And new PSs are developed that can be excited by the corresponding light sources (112), such as phthalocyanines can be excited by longer-wavelength NIR light (113). They commonly have strong energy absorption with large conjugated domains and high fluorescence quantum yield, which is necessary for PDT (114). However, most phthalocyanines are hydrophobic (115). Leandro et al. used nanocarriers, nanoemulsions (ClAlPc-NE) and nanocapsules (ClAlPc-NC) to improve the solubility and bioavailability of phthalocyanines (77). However, the energy gaps of NIR-activated PSs are narrow and the yield of singlet oxygen is reduced (116). In addition, NIR light can only penetrate 5 mm of tissue and needs to retain sufficient energy for PS activation (117). Therefore, PDT of NIR light in prostate cancer is limited. One solution is to utilize upconversion materials combined with PSs that can be excited by NIR. So PSs can be excited by low-energy light, such as in the UV-Vis range (118). Burcu et al. reported Yb/Er (30%: 3%) upconversion nanoparticles (UCNP) made of Y_2_O_3_, Yb_2_O_3_, and Er_2_O_3_. The UCNP modified with a shell of mesoporous silica or gold (Au), then loaded with 2 PSs, merocyanine 540 (MC540) and zinc phthalocyanine (ZnPc) (80). Besides NIR light, X‐rays has received a lot of attention due to the infinite penetrating. However, most PSs do not directly absorb X-ray photons well and require the help of scintillator to convert X-rays into photons that can activate the PSs. Therefore, many studies have reported PSs can be carried by functionalizing the surface of scintillating nanoparticles (119). A few studies about scintillating nanoparticles loaded with PpIX; were reported for prostate cancer, such as a nanocomposite system of Ce^3+^ doped lanthanum (III) fluoride (LaF_3_:Ce^3+^) (82). Prakhar et al. synthesized core-shell nanoparticles. The core was scintillating nanoparticles Y_2.99_Pr_0.01_Al_5_O_12_ (YP), which converted X-ray photons into UVA photons. The sell was mesoporous silica loaded with PpIX and FA (81). Chen et al. reported afterglow (persistent luminescence) nanoparticles, hydrosoluble Sr_2_MgSi_2_O_7_:Eu^2+^, Dy^3+^ coating with (aminopropyl) triethoxysilane (APTES) and conjugate with PpIX and FA (83). Although scintillation nanoparticles combined with PSs have shown great promise for PDT of prostate cancer, their biocompatibility, safety, and clinical feasibility need to be further investigated. In addition, the scintillation nanoparticles have low luminous efficiency and low energy transfer to PS, still no substantial results have been achieved. Another strategy to overcome the depth limitation is to use photons generated by enzyme-mediated bioluminescence approaches to excite the PS instead of an external excitation light source. Recently, a new system that enables intracellular PDT effects with no external light was reported. Coelenterazine, a chemiluminescent single molecule that is widely present in marine organisms. Magalhães et al. utilized the heavy-atom effect to enhance the efficiency of intersystem crossing, then enable the derivatives of coelenterazine generated triplet states (T_1_) by chemiluminescent reaction triggered by O_2_^-•^. Further studies found that the intracellular PDT anticancer activity of these coelenterazine derivatives is more relevant to prostate and breast cancer (84). The development of new self-activating PSs for prostate cancer PDT may be a promising approach.

### Hypoxic strategies

Oxygen is one of the three elements and is an important substance involved in photodynamic action, so the concentration of oxygen in the tissue plays a very important role in the effect of photodynamic. During PDT, tissue oxygen concentration is affected by many factors, such as blood flow, blood oxygenation, etc. The photosensitivity reaction can induce vascular destruction, vasoconstriction, and blood cell retention and agglutination. However, the stagnation of blood flow may also lead to tumor hypoxia during PDT, resulting in decreased efficacy (120). Therefore, many studies have tried to enhance the efficacy of PDT by modulate tumor cell-microenvironment of prostate cancer. One strategy is to deliver oxygen directly to tumors *via* nanoparticles. Huang et al. utilized bone marrow-derived monocytes to escape from immune cell clearance as a carrier to transfer pure oxygen and ce6 (85). Besides PS and oxygen, superparamagnetic iron oxide nanoparticles were also loaded to induced photothermal therapy (PTT) in prostate cancer Tramp-C1 cells. Another strategy is to use an oxygen-independent free radicals to make up the disadvantages of hypoxia environment. Sun et al. synthesis nanoparticles containing indocyanine green (ICG) for PTT and PDT, (2, 2’-azobis[2-(2-imidazolinI-2-yl) propane] dihydrochloride (AIBI) for radical initiator, heat shock protein inhibitors (geldanamycin, 17-AAG) for synergizing PDT. The nanoparticle surface was modification with hyaluronic acid (HA) to target CD44 receptor overexpressed on the surface of prostate cancer cell (86). Ludivine Larue et al. reported a multifunctional platform combining three units: a PS (Pyro-a), a peptide to target tumor neovessels, and an Alkoxyamines (Alks) for an oxygen-independent activity (121). However, this platform has not been tested on cells and animals. This nanoparticle system actually enhanced the efficiency of PDT by synergistic therapy in multiple ways.

## Combination with other therapies

Although PDT has a lot of advantages of high specificity, strong reproducibility, and low invasion (122). It is difficult to eradicate prostate cancer completely by PDT alone, especially following a single course of treatment. The combination of PDT with other therapeutic modalities could provide opportunities to draw on strong points of other therapeutic modality to offset one’s own weaknesses, leading to additive or even synergistic therapeutic effects. Multimodal therapies combine with PDT are promising for multidrug resistance (MDR) and hypoxia-related prostate cancer treatment (**Table 5**).

**Table 5:** Different therapies studies combine with PDT for prostate cancer.

Therapies	Molecular conjugates PS/NPs	Cell model	λ_max_	Animal model	Light dose	Ref.
**PDT/PTT**	PGL-DiR	PC3	(PDT) 650 nm (PTT) 760 nm	Male Balb/C mice	n.a./200 mW cm^-2^ n.a./1 W cm^-2^	(123)
**PDT/PTT**	HSA-ICG-Ce6	PC3	(PDT) 660nm (PTT) 808nm	Nude mice	n.a./0.2 W cm^-2^ n.a./1 W cm^-2^	(124)
**PDT/PTT**	Ce6@PDA-PEG-PFP	LNCaP and PC3	660 nm	5-week-old male Balb/c nude mice	n.a./0.5, 1.5, 5, 40 and 90 mW cm^-2^	(125)
**PDT/PTT**	AuNR/ICG vesicle	PC3	785 nm	Male Balb/c nude mice	n.a./1 W/cm^2^	(126)
**PTT/CT**	SGNS-5-Flouroacil	PC3 and DU145	808 nm	n.a.	n.a./1.8, 2.5 and 3 W cm^-2^	(127)
**PDT/PTT/CT**	HSA@IR780@DTX	22RV1	808 nm	BALB/c nude mice	n.a./1 W cm^-2^	(128)
**PDT/PTT/CT**	PTX- Pluronic-PEI @Au	PC3	808nm	Nude mice	n.a./1 W cm^-2^	(129)
**PDT/CT**	Rose Bengal-MNCs (CTS/PVA/bPEI)	Tramp-C1	532 nm	7-week-old Balb/c mice	n.a./15 and 100 W cm^-2^	(130)
**PDT/PTT/CT**	Dox@PAH-cit/PDA	PC3, DU145 and LNCaP	808nm	Male Balb/c mice	n.a./1.5 W cm^-2^	(131)
**PDT/CT**	IR780-Abiraterone	PC3, DU145, C4-2 and LNCaP	n.a.	Athymic nude mice and 4- to 6-week-old Balb/c mice	n.a.	(132)
**PDT/CT**	PSMA-1-MMAE-IR700	PSMA^+^ PC3pip and PSMA^-^ PC3flu	690 nm	6- to 8-week-old male athymic nude mice	1, 3 and 50 J cm^-2^/n.a.	(96)
**PDT/CT**	TPCI/PTX@ liposomes	PC3	460 nm	Male BALB/c nude mice	n.a./1.0 mW cm^−2^	(133)
**PDT/PTT/CT**	J591-ICG	PSMA^+^ PC3pip and PSMA^-^ PC3flu	785 nm	7- to 8-week-old athymic nu/nu mice	n.a.	(134)
**PIT**	Anti-PSMA mAb/IR700	PSMA^+^ PC3pip-luc and PSMA^-^ PC3flu	689 nm	6- to 8-week-old female homozygote athymic nude mice	50 and 100 J cm^-2^-50 mW cm^-2^	(135)
**PIT**	J591 (IgG, Db, Mb)/IR700	PSMA^+^ PC3pip and PSMA^-^ PC3flu	670-10 nm	6- to 8- week-old female homozygote athymic nude mice	50 and 100 J cm^-2^-25 mW cm^-2^	(136)
**PTT/PIT**	^111^In-DTPA-D2B-IR700	PSMA^+^ LS174T and PSMA^-^ LS174T-wildtype cells	670-710 nm	male BALB/c nude mice	2, 5, 10, 30, 50, 100 and 150 J cm^-2^/n.a.	(137)
**PDT/PTT/CT/PIT**	GNS@IR820/DTX-CD133	PC3	808 nm	4- to 6-week-old male BALB/c athymic nude mice	n.a./0.8 mW cm^-2^	(138)

PGL, porphyrin grafted lipid; DiR, 1-dioctadecyl-3,3,3,3-tetramethylindotricarbocyanine iodide; PTT, Photothermal therapy; CT, chemotherapy; PTX, Paclitaxel; PIT, photoimmunotherapy; mAb, monoclonal antibody; SGNS, Silver gold nanoshell; MMAE, monomethyl auristatin E, CTS, chitosan; PVA, poly (vinyl alcohol); bPEI, branched polyethylenimine; Db, diabody; Mb, minibody.n.a., not applicable.

### Combined PDT with PTT

PTT is another form of phototherapy that converts light energy into heat energy to kill cancer cells (139). When the photothermal agent accumulated in tumors is irradiated by an external light source of a specific wavelength (generally NIR light) (140). Many studies have reported PTT treatment for prostate cancer (141). The combination of PTT and PDT has potential synergistic effects: the thermal effect of PTT is independent of oxygen, which can improve local blood flow and increase the oxygen concentration in tumor tissue, thereby making up for the deficiency of PDT in hypoxic environment and making PDT more effective. In addition, ROS generated during PDT can damage heat shock proteins, thereby reducing their protective effect on tumor cells during PTT. Many studies reported the combination PSs and photothermal agents in the treatment of prostate cancer through nanomaterials as carriers (142). Bhattarai et al. reported self-assembled NPs combined porphyrin grafted lipid (PGL) with cyanine dye DiR for PDT/PTT in prostate cancer (123). The results showed that more ^1^O_2_ was produced when the PGL-DiR NPs were first irradiated by 760 nm followed by a 650 nm and significant synergistic effect on tumor growth inhibition. Ji et al. synthesized a human serum albumin (HSA)-based nanoparticles loaded with Ce6 and ICG for prostate cancer. The results showed ICG could quench the fluorescence of Ce6. Only when the nanoparticles were first received 808 nm light irradiation, ICG degraded and converted light energy into heat energy to kill cancer cells. Followed by 660 nm irradiation, Ce6 produce lots of ^1^O_2_ for PDT (124). Besides photothermal drugs, photothermal nanomaterials have also been used as carriers to link PS for synergistic therapy. Melanin-like polydopamine nanoparticles (PDA) has been used to be a delivery platform due to its excellent photothermal conversion efficiency, biocompatibility, and simple functionalization. Dai et al. reported a multifunctional nanoplatform, PDA loaded with Ce6 and a small-molecule PSMA inhibitor (DCL) was modified with perfluoropentane (PFP). PDA for PTT, Ce6 for PDT, DCL for targeting to prostate cancer and PFP for ultrasound imaging. The nanoplatform demonstrated ultrasound contrast signal at the prostate tumor site and synergistic killing effect on prostate cancer cells (125). The paper does not mention whether the sequence of laser irradiation at 660 nm or 808 nm affects the killing effect. Since the absorption spectra of photothermal and photodynamic agents are usually different, combined PTT and PDT require sequential irradiation of the tumor with two different lasers, which prolongs treatment time and complicates the treatment process. Cui et al. reported a self-assembled nanoplatform could be triggered by a single laser to achieve synergistic PTT and PDT. The inorganic (gold nanorods) and organic phototherapeutic materials (ICG) were irradiated at 785 nm to generate ROS and heat for PDT/PTT (126). However, the excitation of PDT and PTT by the same wavelength light mainly comes from ICG, the ROS production of ICG is limited while Au mainly provides the photothermal effect. In addition, AuNPs can quench some PSs and therefore affect the yield of ^1^O_2_. Therefore, how to maximize the synergistic effect of PDT and PTT remains a lot of questions due to the low photoconversion efficiency.

### Combined PDT or PTT with chemotherapy (CT)

The reproducibility and non-resistance of PDT may provide a new option for drug-resistant patients, while chemotherapy can compensate for the limitation of light penetration in PDT, and may also enhance the sensitivity of cancer cells to ROS or hyperthermia thereby achieving synergistic treatment. Several studies have explored the compatibility of PDT or PTT with CT for prostate cancer. Most of them achieve the delivery of PSs and chemotherapeutic drugs by using nanoparticles (127). IR780, a NIR dye with both photodynamic and photothermal properties. However, the hydrophobicity and toxicity of IR780 limit its translation in the clinic. Lian et al. encapsulated IR780 into human serum albumin (HSA) to improve its hydrophobicity. Docetaxel (DTX), first-line antitumor chemotherapy drug was also encapsulated into NPs for prostate cancer. HSA@IR780@DTX NPs were developed for imaging and PTT/PDT/CT of castration-resistant prostate cancer (128). Wang et al. synthesized a multifunctional NP for CRPC. The cell cycle chemotherapeutic drug paclitaxel (PTX) was encapsulated in the copolymer Pluoronic-PEI (Pluronic-polyethylenimine) nanoparticles and covered with gold cage for PDT/PTT. When the NPs were irradiated at 808 nm, they could inhibit TRPV6 channel and generated ROS, high temperature and released PTX to synergistic killing CRPC PC3 cells (129). In recent years, more nanoparticle carriers have been synthesized that based on the tumor microenvironment to achieve precise drug release. Yeh et al. reported ROS-responsive tripolymer (CTS/PVA/bPEI) coated magnetic nanoclusters (MNCs) loaded with Rose Bengal (RB) for PTX delivery (130). The polyethylenimine (bPEI)-based MNCs encapsulated PTX and RB through electrostatic interaction. When RB was irradiated and generated ROS (143), PTX was released through attraction reduction of ROS-responsive material bPEI to achieve the effect of combined therapy. The pH-responsive NPs were also used as chemotherapeutic drugs carriers. Zhang et al. synthesized core-shell NPs, the core was PDA for PTT, the shell was a pH-responsive charge-reversal polymer, poly(allylamine)-citraconic anhydride (PAH-cit) loaded with doxorubicin (Dox). Dox was released due to the amide hydrolysis of PAH-cit in the acidic environment of tumor, and PDA produced heat to kill the prostate cancer when the NPs were irradiated by 808 nm laser (131). However, the toxicity, poor metabolizability and batch-to-batch variability of NPs greatly limit the clinical translation. Small molecule drugs are more economical and less likely to cause immune reactions than NPs. Abiraterone is a 17α−hydroxylase/C17, 20−lyase (CYP17) inhibitor that has been used in patients with prostate cancer after ADT, which was conjugated with NIR fluorescent dye IR780 for prostate cancer imaging and therapy (132). Monomethyl auristatin E (MMAE) was a chemotherapeutic drug widely used in antibody drug conjugates (ADCs) (144), which was conjugated with IR700 and PSMA-1 for prostate cancer targeted therapy combined with CT and PDT (96). A PS TPCI and PTX were co-encapsulated in liposomes, which exhibited a superb synergistic anticancer effect against prostate cancer. The *in vivo* study showed TPCI/PTX@Lipo could effectively ablate 200 mm^3^ tumor and monitor early tumor response (133).

### Combined PDT or PTT and photoimmunotherapy (PIT)

PTT and PDT can induce immunogenic cell death (ICD) and activate damage-related molecular mechanisms, thereby increasing tumor immunogenicity. Therefore, many studies using ICDs generated from PTT and PDT to enhance the efficacy of immunotherapy have been reported. Monoclonal antibodies are widely used as therapeutic drugs due to targeting specificity. A large number of ADCs have been reported. And they have also been used as carriers of PS to combined with PDT and immunotherapy (134). To improve the specificity of PDT, PSs are conjugated to tumor-specific monoclonal antibodies or single-chain antibody fragments (scFvs), which enables the delivery of PSs to tumor tissues. This method is called photoimmunotherapy (PIT) and was pioneered by Levy et al. in 1983 (145). The first antibody-photosensitizer conjugate (APC) of world is RM-1929, an antibody cetuximab targeting epidermal growth factor receptor (EGFR)-conjugated PS IR700, was approved for marketing in Japan in September 2020 for the treatment of head and neck squamous cell carcinoma. Since most traditional PSs are hydrophobic, many APCs lose specificity and produce toxicity, which limits the development of APCs. The water-soluble PS, silicon phthalocyanine dyes have attracted attention (146). A fully human anti-PSMA mAb-IR700 conjugate was reported (135). After APC was irradiated with NIR light, prostate cancer cells rapidly necrosis, tumor growth was inhibited, and survival rate was improved. Rira Watanabe et al. report the photoimmune efficacy of antibody fragments, including anti-PSMA secondary antibodies (Db) and mini antibodies (Mb), with intact IgG conjugates with IR700. The results demonstrated that equally effective PIT could be achieved with both full antibodies and antibody fragments. However, Db-IR700 conjugate could minimize the time interval between injection and NIR irradiation (136). More multimodal therapies for prostate cancer have been developed. The anti-PSMA mAb D2B labeled with ^111^In and conjugated to the IR700 was used to radionuclide and NIR fluorescence imaging for preoperative and intraoperative detection of PSMA^+^ prostate cancer. Furthermore, the photodynamic response of IR700 was activated to kill the tumor (137). Tan et al. established a multifunctional nanoplatform, gold nanostars (GNS) were coated with PEG, functionalized with CD133 antibody, loading IR820 and DTX for synergistic PTT/PDT/CT treatment of CRPC (138). This multimodal approach integrating diagnosis and treatment may become the future development of APC drugs.

## Conclusions

PDT is a potential, minimally invasive treatment for localized prostate cancer that selectively ablates the disease with minimal effects on functional outcomes than RP, particularly on the bladder, rectum, and neurovascular bundle. Due to oncological safety, low toxicity and support by level 1 evidence from the only RCT within the focal therapy literature, PDT holds significant promise as the focal therapy of choice over other modalities for men with localized prostate cancer. However, there are still a lot of uncertainties and shortcomings. Although prostate cancer is multilocular, existing imaging techniques are unable to detect microscopic lesions throughout the gland. The efficacy of high-risk localized disease is not sufficient with the existing PDT for prostate cancer because they do not target the entire prostate gland. However, with the development of early detection techniques such as liquid biopsy, more prostate cancers are being detected at an early stage. A broader target group will be served by minimally invasive procedures like PDT. Additionally, there are few treatment options available for CRPC and locally recurrent prostate cancer. The potential to create new PDT platforms with enhanced capabilities and more potent therapies for patients with prostate cancer is enormous. To develop a straightforward strategy that can serve as a reference for clinical design by studying the optical characteristics of different types of prostate cancer and the surrounding normal tissue. Develop associated detection and monitoring equipment to control the PDT dose in order to take repeated treatments into consideration. Explore methods to detect the dose or response amount of photodynamic action in the illuminated region during the treatment process. Novel PSs and supporting lasers for prostate cancer treatment will continue to be created with improved photodynamic activity, deeper killing, better selectivity, and less skin adverse effects. To enable fluorescence diagnosis, intraoperative imaging, and therapeutic integration, multimodal PSs and photodiagnostic devices compatible with various medical endoscope systems or interventional devices may be developed.

## Author contributions

WQ and XY contributed to conception and design of the study. QX organized the database. QX wrote the first draft of the manuscript. QX, JJ, and JZ wrote sections of the manuscript. All authors contributed to manuscript revision, read, and approved the submitted version

## Conflict of interest

The authors declare that the research was conducted in the absence of any commercial or financial relationships that could be construed as a potential conflict of interest.

## Publisher’s note

All claims expressed in this article are solely those of the authors and do not necessarily represent those of their affiliated organizations, or those of the publisher, the editors and the reviewers. Any product that may be evaluated in this article, or claim that may be made by its manufacturer, is not guaranteed or endorsed by the publisher.

## Abbreviations

WHO, World Health Organization; IARC, International Agency for Research on Cancer; PDT, Photodynamic Therapy; PS, Photosensitizer; PSA, prostate-specific antigen; MRI, Magnetic Resonance Imaging; RP, radical prostatectomy; EBRT, external beam radiation therapy; ADT, androgen deprivation therapy; ROS, reactive oxygen species; FDA, U.S. Food and Drug Administration;NMDA, National Medical Products Administration;EMA, European Medicines Agency; NSCLC, non-small cell lung cance; HP, Hematoporphyrin; HpD, Hematoporphyrin derivative; mTHPC, m-tetra-hydroxyphenyl chloride; Mlu, Motexafin Lutetium; ALA, aminolaevulinic acid; Pp IX, protoporphyrin; LED, light emitting diode; TRUS, transrectal ultrasound; NPs, Nanoparticles; Ptl, Tetrasulfonated aluminum phthalocyanine; FNP, fluorescent nanoparticles; Ppa, Pyropheophorbide-A; PSMA, Prostate-specific membrane antigen; PARP, polyADP-ribose polymerase; PSMA-1, PSMA ligand (Glu-CO-Glu′-Amc-Ahx-Glu-Glu-Glu-Lys-NH2); AuNPs, gold nanoparticles; PFP, perfluoropentane; PTT, photothermal therapy; RGD, arginine-glycine-aspartic acid; c(RGDyK), a cyclic RGD peptide; PTA, Polyamine Transport Systems; FA, folic acid; FR, folate receptor; CI-M6PR, Cation-independent mannose 6-phosphate receptor; LC-Pyro, Long-circulating Pyropheophorbide; BChl, Bacteriochlorophyll; PA, Polyamine; MSNs, Mesoporous silica nanoparticles; M6C, Mannose 6-Carboxylate; M6C-Man, dimannoside-carboxylate; NE, Nanoemulsions; NC, Nanocapsules; HVJ-E, Hemagglutinating virus of Japan envelope; UV, ultraviolet; UCNP, Upconversion nanoparticles; YPMS, Y2.99Pr0.01Al5O12 afterglow; AG, Sr2MgSi2O7:Eu2+, Dy3+ afterglow; PGL, porphyrin grafted lipid; DiR, 1-dioctadecyl-3,3,3,3-tetramethylindotricarbocyanine iodide; PTT, Photothermal therapy; CT, chemotherapy; PTX, Paclitaxel; PIT, photoimmunotherapy; SGNS, Silver gold nanoshell; MMAE, monomethyl auristatin E; ICG, indocyanine green; AIBI, (2,20-azobis[2-(2-imid- azolinI-2-yl) propane] dihydrochloride; 17-AAG, heat shock protein 90 inhibitor geldanamycin; HA, Hyaluronic acid; MDR, multidrug resistance; HAS, human serum albumin; CYP17, 17α−hydroxylase/C17, 20−lyase; DTX, docetaxel; ADC, antibody drug conjugate; bPEI, polyethylenimine; CT, chemotherapy; ICD, immunogenic cell death; PIT, photoimmunotherapy; NIR-PIT, Near-infrared photoimmunotherapy; APC, mAb-photoabsorber conjugate; Db, diabody; Mb, minibody.

## Glossary

**Table:**

17-AAG	heat shock protein 90 inhibitor geldanamycin
ADT	androgen deprivation therapy
ALA	aminolaevulinic acid
APC	mAb-photoabsorber conjugate
AuNPs	gold nanoparticles
AG	Sr2MgSi2O7:Eu2+, Dy3+ afterglow
AIBI	(2,20-azobis[2-(2-imid- azolinI-2-yl) propane] dihydrochloride
ADC	antibody drug conjugate
bPEI	polyethylenimine
BChl	Bacteriochlorophyll
c(RGDyK)	a cyclic RGD peptide
CI-M6PR	Cation-independent mannose 6-phosphate receptor
CT	chemotherapy
CYP17	17α−hydroxylase/C17, 20−lyase
CT	chemotherapy
DTX	docetaxel
DiR	1-dioctadecyl-3,3,3,3-tetramethylindotricarbocyanine iodide
Db	diabody
EBRT	external beam radiation therapy
EMA	European Medicines Agency
FDA	U.S. Food and Drug Administration
FNP	fluorescent nanoparticles
FA	folic acid
FR	folate receptor
HA	Hyaluronic acid
HVJ-E	Hemagglutinating virus of Japan envelope
HAS	human serum albumin
HP	Hematoporphyrin
HpD	Hematoporphyrin derivative
ICD	immunogenic cell death
IARC	International Agency for Research on Cancer
ICG	indocyanine green
LC-Pyro	Long-circulating Pyropheophorbide
LED	light emitting diode
MSNs	Mesoporous silica nanoparticles
M6C	Mannose 6-Carboxylate
MMAE	monomethyl auristatin E
M6C-Man	dimannoside-carboxylate
mTHPC	m-tetra-hydroxyphenyl chloride
Mlu	Motexafin Lutetium
MDR	multidrug resistance
Mb	minibody
MRI	Magnetic Resonance Imaging
NMDA	National Medical Products Administration
NSCLC	non-small cell lung cance
NPs	Nanoparticles
NIR-PIT	Near-infrared photoimmunotherapy
NE	Nanoemulsions
NC	Nanocapsules
Ptl	Tetrasulfonated aluminum phthalocyanine
Ppa	Pyropheophorbide-A
PSMA	Prostate-specific membrane antigen
PARP	polyADP-ribose polymerase
PSMA-1	PSMA ligand (Glu-CO-Glu′-Amc-Ahx-Glu-Glu-Glu-Lys-NH2)
PA	Polyamine
PTT	Photothermal therapy
PFP	perfluoropentane
PTX	Paclitaxel
PIT	photoimmunotherapy
PTT	photothermal therapy
PGL	porphyrin grafted lipid
PTA	Polyamine Transport Systems
PIT	photoimmunotherapy
PDT	Photodynamic Therapy
PS	Photosensitizer
PSA	prostate-specific antigen
PpIX	protoporphyrin IX
ROS	reactive oxygen species
RP	radical prostatectomy
RGD	arginine-glycine-aspartic acid
SGNS	Silver gold nanoshell
TRUS	transrectal ultrasound
UV	ultraviolet
UCNP	Upconversion nanoparticles
YPMS	Y2.99Pr0.01Al5O12 afterglow
WHO	World Health Organization
